# Defining the regenerative effects of native spider silk fibers on primary Schwann cells, sensory neurons, and nerve‐associated fibroblasts

**DOI:** 10.1096/fj.202001447R

**Published:** 2020-11-19

**Authors:** Flavia Millesi, Tamara Weiss, Anda Mann, Maximilian Haertinger, Lorenz Semmler, Paul Supper, Dietmar Pils, Aida Naghilou, Christine Radtke

**Affiliations:** ^1^ Research Laboratory of the Division of Plastic and Reconstructive Surgery Department of Surgery Medical University of Vienna Vienna Austria; ^2^ Austrian Cluster for Tissue Regeneration Vienna Austria; ^3^ Division of General Surgery Department of Surgery Comprehensive Cancer Center Vienna Medical University of Vienna Vienna Austria; ^4^ Division of Plastic and Reconstructive Surgery Department of Surgery Medical University of Vienna Vienna Austria

**Keywords:** live cell imaging, migration, *Nephila edulis*, peripheral nerve regeneration, proliferation

## Abstract

The search for a suitable material to promote regeneration after long‐distance peripheral nerve defects turned the spotlight on spider silk. Nerve conduits enriched with native spider silk fibers as internal guiding structures previously demonstrated a regenerative outcome similar to autologous nerve grafts in animal studies. Nevertheless, spider silk is a natural material with associated limitations for clinical use. A promising alternative is the production of recombinant silk fibers that should mimic the outstanding properties of their native counterpart. However, in vitro data on the regenerative features that native silk fibers provide for cells involved in nerve regeneration are scarce. Thus, there is a lack of reference parameters to evaluate whether recombinant silk fiber candidates will be eligible for nerve repair in vivo. To gain insight into the regenerative effect of native spider silk, our study aims to define the behavioral response of primary Schwann cells (SCs), nerve‐associated fibroblasts (FBs), and dorsal root ganglion (DRG) neurons cultured on native dragline silk from the genus *Nephila* and on laminin coated dishes. The established multi‐color immunostaining panels together with confocal microscopy and live cell imaging enabled the analysis of cell identity, morphology, proliferation, and migration on both substrates in detail. Our findings demonstrated that native spider silk rivals laminin coating as it allowed attachment and proliferation and supported the characteristic behavior of all tested cell types. Axonal out‐growth of DRG neurons occurred along longitudinally aligned SCs that formed sustained bundled structures resembling Bungner bands present in regenerating nerves. The migration of SCs along the silk fibers achieved the reported distance of regenerating axons of about 1 mm per day, but lacked directionality. Furthermore, rFBs significantly reduced the velocity of rSCs in co‐cultures on silk fibers. In summary, this study (a) reveals features recombinant silk must possess and what modifications or combinations could be useful for enhanced nerve repair and (b) provides assays to evaluate the regenerative performance of silk fibers in vitro before being applied as internal guiding structure in nerve conduits in vivo.

**Abbreviations**
DRG: dorsal root ganglion
ECM: extracellular matrix
FB: fibroblast
PDL: poly‐d‐lysine
PLL: poly‐l‐lysine
PNI: peripheral nerve injury
SC: Schwann cell

## 1 INTRODUCTION

Successful restoration of nerve function after peripheral nerve injuries (PNIs) remains one of the biggest challenges in regenerative medicine.^1^, ^2^, ^3^, ^4^, ^5^ Despite continuous efforts to improve recovery by surgical techniques and physiotherapeutic measures, follow‐up surgeries and residual manifestation of nerve dysfunction severely impair the patients’ quality of life.^6^

Remarkably, the peripheral nervous system is capable to self‐regenerate after minor PNIs, such as crush injuries, when most of the nerves’ connective tissue remains intact.^7^ This regenerative potential is attributed to Schwann cells (SCs), which are able to adapt a specialized repair phenotype after PNI.^8^ Repair SCs acquire specific functions that are essential for the multi‐step process of nerve regeneration such as the removal of myelin debris, the attraction of immune cells, and neurotrophic support for injured axons.^9^, ^10^, ^11^, ^12^ In addition, repair SCs proliferate and align within their basal lamina tube, surrounding each SC‐axon unit, to form Bungner bands.^11^, ^13^ These regeneration tracks are important for nerve repair as they provide neuritogenic cues and orientation for re‐growing axons.^11^ Severe PNIs including nerve transection or tissue loss disrupt the nervous architecture and usually exceed the peripheral nervous systems’ inherent regenerative capabilities. Then, SCs face the challenge of directing axons without the guidance of their basal lamina tubes, which commonly results in disorganized axon sprouting, neuroma formation, and pain. As a consequence, the distal nerve end experiences long‐term denervation leading to target organ atrophy and functional loss.^14^, ^15^, ^16^

In order to treat severe PNIs, different surgical procedures have been established.^17^ A straight transection usually allows tension free end‐to‐end nerve coaptation.^18^ The current gold standard to replace lost nerve tissue is the transplantation of an autologous nerve graft.^17^, ^19^, ^20^ However, the excision of autologous nerve tissue entails functional loss of the innervated donor site and bares a considerable risk of donor site morbidity.^21^, ^22^ Thus, research has provided other strategies to repair severely damaged nerves.^23^, ^24^ A tempting alternative to obviate the need for nerve autografts are off‐the‐shelf available artificial nerve conduits. These hollow tubes mimic the nerves outermost layer, the epineurium, and offer a simple guiding channel for the regenerating nerve and protection from scar tissue‐forming fibroblasts.^25^ Remarkable advances in the field of tissue engineering led to the development of a variety of nerve conduits.^3^, ^23^, ^26^, ^27^ Many of them achieved regenerative effects comparable to nerve autografts in short‐distance nerve gaps of 1‐3 cm,^28^, ^29^ however, they are still inferior to the autograft in large‐distance nerve gaps > 3 cm.^28^, ^30^, ^31^ Long hollow conduits may collapse or fail to assist the re‐growing nerve due to the lack of a nervous architecture that usually provides optimal conditions for nerve recovery in form of cells, signaling molecules, and the extracellular matrix (ECM).^32^, ^33^ Hence, a promising approach to enhance the performance of artificial nerve conduits is their enrichment with an internal framework that (a) improves the microenvironment by neuritogenic and neurotrophic support, (b) promotes the survival and regenerative functions of cells, and (c) offers orientation and guidance for the re‐growing nerve.^33^, ^34^, ^35^ The ideal material should be biocompatible, resilient, biodegradable, and, importantly, allow cell attachment, migration, and proliferation.^36^ Both synthetic and biological materials have found their use in nerve tissue engineering. They include luminal fillers such as hydrogel‐forming matrices, and internal guiding structures such as micro‐channels or longitudinally aligned filaments.^27^, ^35^, ^37^, ^38^ However, synthetic polymers often lack elasticity, poorly integrate into the native tissue, which is linked to chronic inflammation, tissue fibrosis, and scarring that result in a physical barrier for regenerating axons.^39^, ^40^, ^41^, ^42^ The material inserted into the lumen of nerve conduits should, thus, prevent a fibrous tissue reaction evoked by immune cells and a deregulated fibroblast activation characterized by proliferation and extensive ECM deposition.^43^, ^44^ Natural polymers provide biological binding sites for cells and show superior biocompatibility and biodegradability,^45^ but their clinical use is hampered by their relative expense, degradation kinetics, and limited mechanical properties.^46^

Previous studies have investigated the application of silks in nerve regeneration due to their superior mechanical properties.^40^, ^47^, ^48^, ^49^, ^50^ First experiments involved the cocoon silk produced by the larvae of the domestic silk moth *Bombyx mori* that is composed of two major proteins, fibroin, and sericin.^51^ However, a prerequisite for its use is the complete removal of sericin, also called degumming, as sericin was reported to elicit an inflammatory response in vivo.^52^, ^53^ Therefore, research focused on spider silks that evolved independently from insects and possess advantageous properties.^54^ Most studies utilized the dragline silk fiber of the genus *Nephila* ^40^, ^47^, ^48^, ^49^, ^50^ that is mainly composed of spidroin proteins.^55^ In contrast to *Bombyx mori* cocoon silk, the spiders’ dragline silk lacks the sericin protein, which omits the need for a degumming procedure. First in vitro studies demonstrated that spider silk fibers allow the adhesion and distribution of SCs, neuronal NT2 cells, and NIH/3T3 fibroblasts.^48^, ^56^, ^57^ When used in vivo, spider silk hardly provoked an immune response and showed long‐term degradability.^40^, ^58^ Remarkably, decellularized veins enriched with longitudinally aligned spider silk fibers resulted in a regenerative outcome similar to autologous graft controls after long‐distance nerve defects in rats and sheep.^47^, ^59^ These findings indicate spider silk fibers as intriguing internal guiding material to improve artificial conduits for the treatment of critical nerve defects.

Nevertheless, the clinical use of native spider silk is restricted by its limited availability, time‐consuming harvest and, as every biological material, variability.^46^, ^60^ With respect to future biomedical applications, current research is focusing on the controlled production of silk fibers, for example, derived from recombinant silk proteins.^60^, ^61^, ^62^, ^63^, ^64^ The ultimate goal is to create a recombinant silk fiber that emulates the peerless properties of its native counterpart. Recombinant silk shall not only possess the unique material characteristics, but also show the same (or superior) effect on cells. Previous studies suggest different methods to analyze the behavior of cells to silk fibers^57^, ^63^, ^65^, ^66^, ^67^ or silk‐based materials^68^, ^69^ in vitro. However, quantitative and qualitative read‐outs designed to assess the biological response of cells to silk fibers concerning nerve tissue regeneration are scarce. Thus, the features recombinant silk should exhibit before being considered for nerve repair remain insufficiently described.

To accelerate the implementation of recombinant silk fibers as internal guiding structure for nerve conduits, it is important to first understand how native spider silk supports the regeneration of nerves. Herein, we set out to analyze and quantify the regenerative effects that native spider silk exerts on cells involved in nerve repair in vitro and provide a basis for the future production of recombinant silk. In detail, we evaluated to which extent native spider silk replaces the function of the ECM, whose components build structural and informative support important for cellular adhesion, proliferation, and migration during nerve regeneration. The effective re‐growth of injured axons is essential and primarily dependent on the intimate contact to SCs, their key interaction partners.^11^, ^12^, ^70^ Moreover, nerve‐associated fibroblasts (FBs) can positively influence SC behavior,^71^, ^72^ and also impede nerve regeneration by fibrotic tissue formation.^43^, ^73^ Therefore, we established nerve repair‐relevant assays using multi‐color immunofluorescence, confocal microscopy, and live cell imaging to comprehensively characterize the biological behavior of primary SCs, nerve‐associated fibroblasts FBs, as well as dorsal root ganglion (DRG) neurons cultured on native *Nephila* dragline silk fiber meshes or on laminin, a major component of the basal lamina. Our results provide insight into the individual cellular response to native spider silk fibers and facilitate to adjust the properties of recombinant silk fibers to the needs of regenerating nerves.

## 2 MATERIALS AND METHODS

### 2.1 Animals

*Rats:* For this study, outbred Sprague‐Dawley rats (stock Him:OFA) were used. Sciatic nerve tissue and dorsal root ganglia (DRG) were harvested from female and male adult rat cadavers for Schwann cell (rSC), fibroblast (rFB), and rDRG neuron isolation. The sacrifice of animals was conducted in compliance with the Austrian's Animal Testing Law (TVG 2012, §2, 1.c) and Article 3 of the Directive 2010/63/EU of The European Parliament and of the Council on the Protection of Animals Used for Scientific Purposes.^74^

*Spiders:* Spiders of the species *Nephila eduli*s were kept individually in glass terraria at about 25°C and 60%‐80% humidity. Webs were sprayed with water every day and the spiders were fed crickets (*Acheta domesticus*) twice a week.

### 2.2 Dragline silk harvest

Harvesting of the dragline silk fibers from the major ampullate gland of adult female spiders was performed as described before.^58^ The fibers were arranged in a crisscross pattern around weaving metal frames of about 1x1 cm^2^ that were bent from stainless steel wire (Remanium, Ø 0.70 mm) using Aderer three jaw pliers (DENTAURUM). Before cell seeding, the silk frames were sterilized in 70% ethanol for 10 minutes and left to dry for at least 30 minutes.

### 2.3 Isolation and culture of rSCs and rFBs

Primary rSC and rFB cultures: Excised rat sciatic nerves were washed in 1× Dulbecco's Phosphate‐Buffered Saline (1× PBS, GIBCO) + 1% antibiotic‐antimycotic (ThermoFisher). The following procedure was performed according to previous studies with some modifications.^75^, ^76^ Briefly, the collected nerve fascicles were digested overnight in MEM∝ (GIBCO) supplemented with 10% fetal calf serum (FCS, LINARIS), 1% Penicillin‐Streptomycin (P/S, GIBCO), 1% Sodium Pyruvate Solution (GIBCO), 2.5% 4‐(2escribed.^12^, ^76^ Briefly, the nerve digest was seeded on 0.01% poly‐L‐lysine hydrobromide (PLL) (SIGMA‐Aldrich) and 4 µg/mL laminin (SIGMA) coated dishes in culture medium consisting of MEM ∝ supplemented with 1% P/S, 1% Sodium Pyruvate Solution, 2.5% HEPES, 0.5% N‐2 Supplement (GIBCO), 2 μM forskolin (SIGMA‐Aldrich), 10 ng/mL recombinant Heregulinβ‐1 (PeproTech), 10 ng/mL recombinant FGF‐basic (PeproTech), 5 ng/mL PDGF‐ AA (PeproTech), and 5% FCS. Medium was changed three times a week. To separate rFBs from rSCs, we took advantage of the different adhesion properties of SCs and FBs and used the two‐step enrichment procedure esta‐hydroxyethyl)‐1‐piperazineethanesulfonic acid buffer solution (HEPES, SIGMA), 0.125% (w/v) collagenase type IV (GIBCO), 1.25 U/mL Dispase II (Sigma‐Aldrich) and 3 mM Calcium chloride (Merck) at 37°C and 5% CO_2_. rSCs and rFBs were isolated according to an adapted protocol as previously dblished by Weiss et al^76^ resulting in a culture purity of about 95%. rFBs were cultured in in MEM ∝ supplemented with 10% FCS, 1% P/S, 1% Sodium Pyruvate Solution, and 2.5% HEPES on uncoated dishes. rSCs were passaged upon reaching 80%‐90% confluency, rFBs were passaged upon reaching about 80% confluency. rSC and rFB cultures from passage 2 (p2) but not higher than p6 were used for experimentation.

*Primary rDRG neuron cultures: For isolation of* rDRGs, the rat spinal cord was exposed using a Liston bone cutting forceps. The lumbar DRG pairs (L1‐L6) were excised, transferred to 1xPBS with 1% P/S and transported on ice. The isolation and culture of rDRG neurons was performed using an adapted protocol based on previous studies.^77^, ^78^ Under the laminar flow hood, the rDRGs were cut into small pieces and digested with the same digestion solution as used for the nerve fascicle tissue (see above). On the next day, the rDRG digest was centrifuged at 200*g* for 5 minutes and resuspended in 1 mL Neurobasal‐A medium (GIBCO). Then, the DRG solution was carefully put on 1 mL of 20% Percoll solution (Merck) followed by centrifugation at 450*g* for 8 minutes. The pellet was then cautiously washed with 1 mL Neurobasal‐A medium and centrifuged at 1230 *g* for 2 minutes. Finally, the pellet was resuspended in 1 mL rDRG culture medium consisting of Neurobasal‐A medium supplemented with 10 ng/mL recombinant NGF (Invitrogen), 1× B27 supplement (Invitrogen), 2mM L‐glutamine (Invitrogen), and 1% P/S. rDRG neurons were cultured on 0.01% poly‐d‐lysine (PDL, Sigma‐Aldrich) and 4 µg/mL laminin coated dishes. The medium was changed three times a week.

Phase contrast images of rSC, rFB, and rDRG neuron cultures were regularly taken with a benchtop microscope (NIKON Eclipse Ts2R).

For marker expression analysis via immunofluorescence, 0.8 × 10^4^ rSCs, 0.8 × 10^4^ rFBs and about 50 rDRG neurons were grown in 8‐well chambers (ibidi). To compare the growth of rSCs on silk versus controls, 1.5 × 10^4^ rSCs were seeded onto a spider silk mesh and a PLL/laminin coated 2‐well chamber (ibidi). To compare the growth of rFBs on silk versus controls, 1.5 × 10^4^ rFBs were seeded onto a spider silk mesh and a PLL/laminin coated 2‐well chamber. Note that cultures on PLL/laminin were subcultured upon reaching 80%‐90% confluency, while cultures on silk were not. To compare the growth of rDRG on silk versus controls, about 50 rDRGs were seeded onto a spider silk mesh and a PDL/laminin coated 2‐well chamber. The cultures were monitored for 20‐30 days.

### 2.4 Proliferation assay

For the proliferation assay, 10 µM 5‐ethynyl‐2'deoxyuridine (EdU, Invitrogen) was added to the cultures and incubated for 20 hours. EdU is a thymidine analog that is incorporated into DNA during the S‐Phase of the cell cycle. Via covalent cross‐linking of a fluorescent azide to EdU (click‐reaction) newly synthesized DNA can be visualized. EdU detection was performed after the immunofluorescence staining procedure (see below) using Click‐iT Plus EdU Alexa Fluor 555 imaging kit (Invitrogen) according to the manufacturer's protocol.

### 2.5 Immunofluorescence staining analysis

All antibody details are listed in Table S1. The procedure was carried out at RT unless otherwise noted. Washing was performed twice after each incubation step, except blocking, with 1×PBS for 5 minutes. Cells were washed and fixed with 4.5% formaldehyde solution (SAV Liquid Production GmbH) for 20 minutes. The rSC characterization staining panels included (a) S100, vimentin (VIME) and DAPI, (b) S100, SOX10, and DAPI and (c) NGFR, THY1, and DAPI. The rFB characterization staining panel included NGFR, THY1 and DAPI. The SC proliferation staining panel included S100, VIME, EdU and DAPI. The rFB proliferation staining panels included THY1, NGFR, EdU, and DAPI. For staining of membrane bound surface proteins, cells were treated with a blocking solution consisting of 1 × PBS containing 1% BSA and 5% goat serum (DAKO) for 30 minutes. The cells were then incubated with the respective primary antibodies in 1 × PBS containing 1% BSA and 1% goat serum followed by washing and incubation with the corresponding secondary antibodies. For staining of intracellular proteins, blocking and permeabilization was performed with 1 × PBS containing 1% BSA, 0.3% TritonX‐100 (SIGMA) and 5% goat serum for 10 minutes. The cells were then incubated with the respective primary antibodies in 1 × PBS containing 1% BSA, 0.1% TritonX‐100 (SIGMA) and 1% goat serum followed by washing and incubation with the corresponding secondary antibodies. EdU detection was performed according to the manufacturer's protocol. For DNA staining, 1 × PBS + 50 μg/mL 4',6‐Diamidino‐2‐Phenylindole (DAPI, ThermoScientific) was added to the cells for 10 minutes. FluoromountG (Invitrogen) was used as mounting medium. The stained cells can be stored at 4°C for up to 2 weeks. Pictures of stained cells were taken using a laser scanning confocal microscope (LEICA SP8X).

### 2.6 Manual counting of cells

For the quantification of (proliferating) rSCs and rFBs, the CellCounter plugin of ImageJ 1.47 (http://imagej.nih.gov/ij/) was used. At least 300 DAPI^+^ nuclei were counted per experimental condition, and burst nuclei and nuclei cut by the image boarder were excluded, which resulted in the total number of real cells. In the rSC culture, S100^+^/VIME^+^ rSCs and S100^+^/VIME^+^/EdU^+^ proliferating rSCs as well as S100^−^/VIME^−^ rFBs, S100^−^/VIME^+^/EdU^+^ proliferating rFBs were determined. In the rFB cultures, THY1^+^/NGFR^−^ rFBs, THY1^+^/NGFR^−^/EdU^+^ proliferating rFBs as well as THY1^−^/NGFR^+^ rSCs and THY1^−^/NGFR^+^/EdU^+^ proliferating rSCs were determined.

### 2.7 Live cell imaging

For live cell imaging, 2‐well chamber slides (Ibidi) were used. Therefore, 1.5 × 10^4^ rSCs or 1.5 × 10^4^ rFBs were seeded in a PLL/laminin coated 2‐well chamber and on a spider silk mesh placed in the other, uncoated 2‐well chamber. To discriminate rSCs from rFBs in co‐cultures on silk, the rFBs were labeled with PKH67 (Sigma‐Aldrich) according to the manufacturer's protocol and 13.5 × 10^2^ rSCs + 1.5 × 10^2^ PKH67‐labeled rFBs were pooled and seeded per spider silk mesh. One hour after seeding, live cell imaging was performed using an Olympus IX83 microscope equipped with *cellSens* live cell imaging software (Olympus Corporation). A phase contrast and a fluorescence picture of three sections per condition were taken every ten minutes for 22 hours. The generated videos and.tiff stacks were analyzed with ImageJ. The *Manual Tracking* plugin was used to manually track 20 rSCs and 20 rFBs per condition throughout the 132 pictures. To prevent a biased picking of cells for analysis, the tracked cells were randomly chosen before the video was evaluated. Afterward, the results were evaluated with the ibidi *Chemotaxis and Migration Tool,* which allowed the calculation of the mean velocity in μm/min and the distance covered by each cell in μm.

### 2.8 Statistical analysis

Data were logarithmized and the significance of differences of velocity, distance, purity, and proliferation values between experimental conditions (CTRL, silk, and silk co‐culture) were estimated with R‐package multcomp 1.4‐12^79^ using a two‐way ANOVA approach followed by Tukey all‐pair comparisons between group means, correcting for the information of the individual cell donors. Two‐way ANOVA allows the estimation of the impact of two different categorical independent variables on one continuous dependent variable, in this case the impact of the experimental condition and the cell donors on the velocity, distance, purity, or proliferation, thus, showing the differences between the individual experimental conditions independent of the cell donors. The data are depicted as single values for each donor ± SD using GraphPad Prism 8.

## 3 RESULTS

### 3.1 Establishment and characterization of rSC, rFB, and rDRG neuron cultures

**FIGURE 1:**
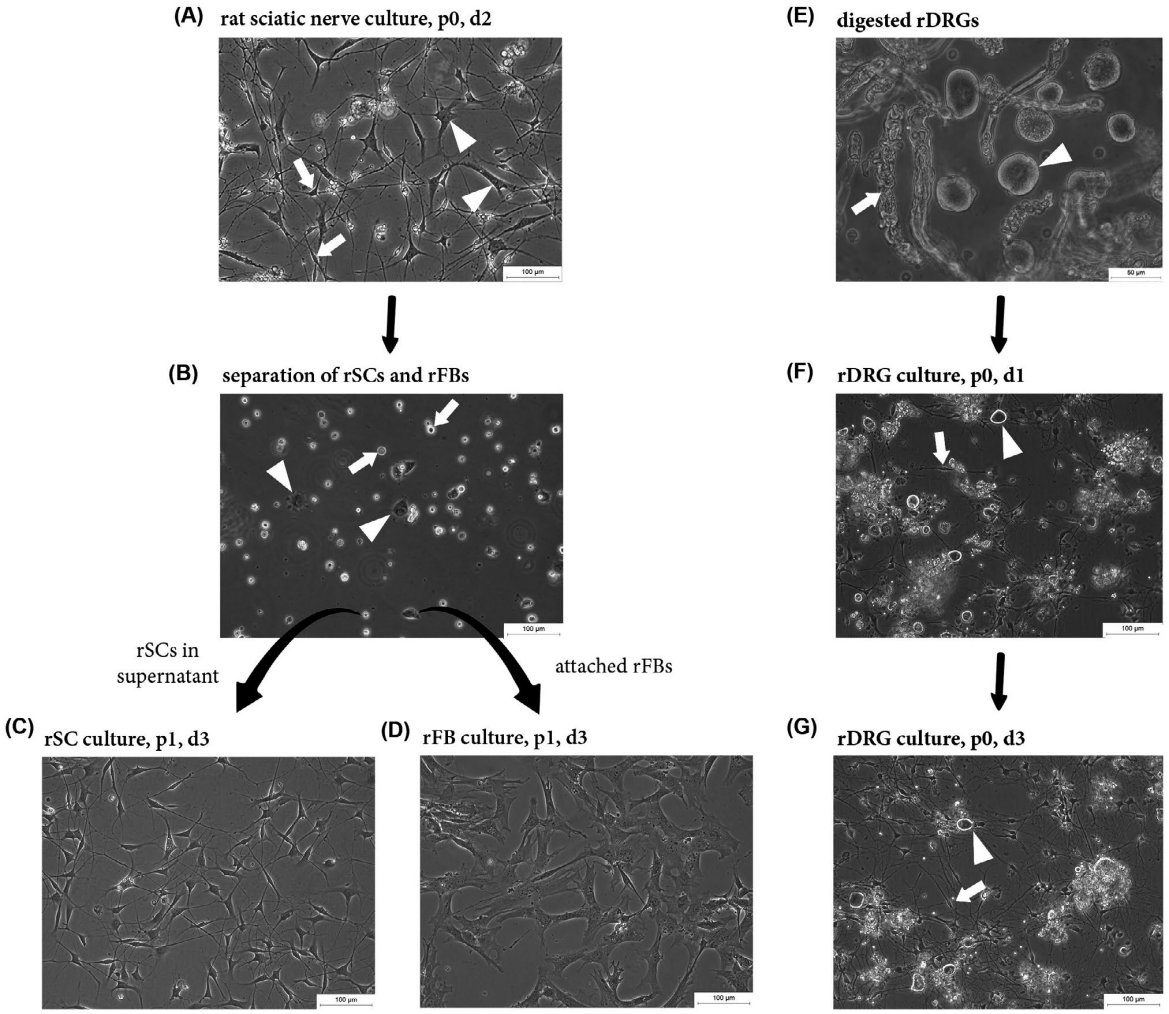
Establishment of rSC, rFB, and rDRG neuron cultures. Representative phase contrast images illustrating the culture and enrichment of rSCs and rFBs (A‐D) and the culture of rDRGs (E‐G). A, A passage 0 (p0) nerve culture consisting of rSCs and rFBs at day 2 after seeding. The distinct morphology between SCs (arrows) and FBs (arrowheads) is evident. B, An uncoated culture dish 30 minutes after plating of p0 cultures with adhered rFBs (arrowheads) and floating rSCs (arrows). C, Enriched p1 rSC culture grown on PLL/laminin with typical bi‐ to tri‐polar extensions. D, An enriched culture of rFBs with typical flattened morphology. E, Digested rDRGs with prominent rDRG neuron cell bodies (arrowhead) and myelin debris (arrow). F,G, p0 rDRG culture grown on PDL/laminin with visible rDRG neurons (arrowhead) and rSCs (arrow) at day 1 (F) and day 3 (G) after seeding

In order to generate primary rSC and rFB cultures for experimentation, the following culture procedure was established. The sciatic nerve tissue was digested and seeded on PLL/laminin coated dishes. Two days after seeding, cell outgrowth was visible and passage 0 (p0) cultures contained rSCs with typical bi‐ to multi‐polar extensions (Figure 1A, arrows) as well as rFBs recognized by a more spread and flattened morphology (Figure 1A, arrowheads). When p0 cultures reached a confluency of about 70%‐80%, rSCs were separated from rFBs by exploiting their different adhesion properties to plastic.^12^ 30 minutes after plating detached p0 cells on uncoated culture dishes, most rFBs already adhered (Figure 1B, arrowheads) while rSCs remained in suspension (Figure 1B, arrows). The floating rSCs were harvested and seeded on PLL/laminin culture dishes. The attached rFBs were further cultured in the uncoated dishes. This procedure enabled to achieve highly enriched rSC (Figure 1C) and rFB (Figure 1D) cultures of >95% purity.^75^, ^76^ To receive primary cultures of sensory neurons, excised rDRGs were digested and the cell suspension (Figure 1E) was grown on PDL/laminin coated dishes. The p0 rDRG cultures consisted of rDRG neurons (Figure 1F, arrowhead), rSCs (Figure 1F, arrow) as well as rFBs and neurite outgrowth was observed 1 day after seeding. After 3 days in culture, rDRG neurons had formed a network of axonal processes with several branches accompanied by rSCs (Figure 1G).

**FIGURE 2:**
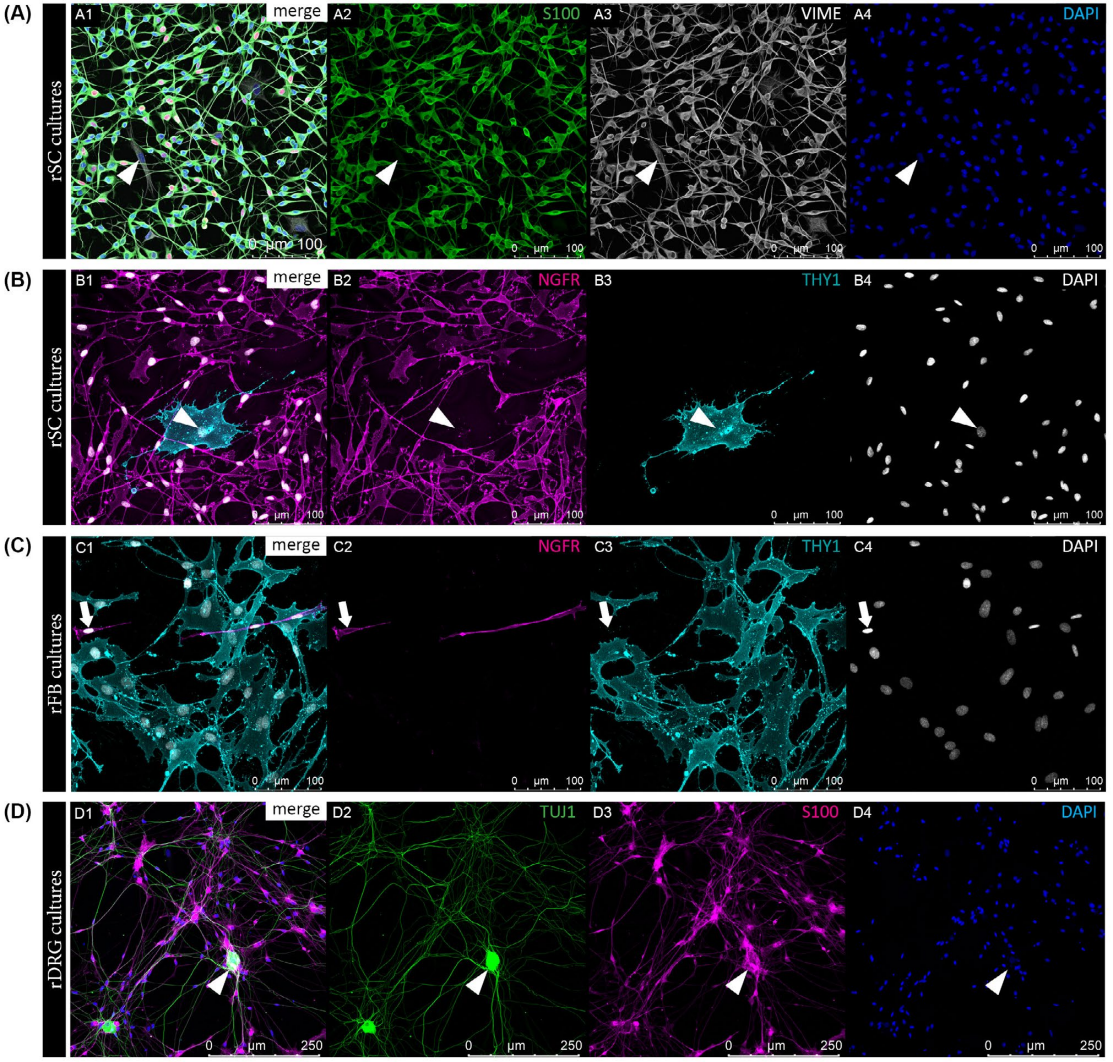
Qualitative characterization of rSC, rFB, and rDRG neuron cultures. Representative immunofluorescence (IF) images of rSC cultures (A‐B), rFB cultures (C), and rDRG cultures (D) illustrating the successful establishment of cell‐specific staining panels. A, IF staining results for rSC cultures with merged channels (A1) and single channels for SC marker S100 in green (A2), VIME in grey (A3) and DAPI positive cell nuclei in blue (A4); arrowheads indicate a S100^−^/VIME^+^ rFB. B, IF staining results for rSC cultures with merged channels (B1) and single channels for SC marker NGFR in magenta (B2), FB marker THY1 in cyan (B3) and DAPI in grey (B4); arrowheads indicate a NGFR^−^/THY1^+^ rFB. C, IF staining results for rFB cultures with merged channels (C1) and single channels for NGFR in magenta (C2), THY1 in cyan (C3) and DAPI in grey (C4); arrows indicate a NGFR^+^/THY1^−^rSC. D, IF staining results for a rDRG neuron culture with merged channels (D1) and single channels for axon associated marker TUJ1 (beta‐3‐tubulin) in green (D2), S100 in magenta (D3) and DAPI in blue (D4); arrowheads indicate a rDRG cell body

The established rSC, rFB, and rDRG neuron cultures were then characterized by the expression of associated markers using immunostainings. To discriminate the different cell types present within the respective cultures, multi‐color staining panels were developed. Confocal imaging of rSC cultures illustrated a high culture purity determined by the expression of the SC‐typic calcium binding protein S100, while both rSCs and rFBs were positive for the intermediate filament vimentin (VIME) (Figure 2A). Another SC‐specific marker upregulated in cultured rSCs is NGFR (low affinity nerve growth factor receptor, also known as p75^NTR^), which showed a strong membranous staining on rSCs (Figure 2B). Accordingly, rFBs were negative for NGFR but expressed the FB‐associated marker THY1 on their cell membrane (Figure 2B). Immunostainings confirmed that the enrichment procedure resulted in a predominant THY1 positive (THY1^+^) rFB population (Figure 2C). Furthermore, rDRG neuron cell bodies and their branched processes showed strong staining signals for β‐3‐tubulin (TUJ1), a major constituent of microtubules important for axon guidance and maintenance (Figure 2D). rDRG cultures also contained S100 positive (S100^+^) rSCs that primarily co‐localized with the axonal processes (Figure 2D). These results demonstrate the successful establishment of rSC, rFB, and rDRG neuron cultures alongside with cell type‐specific multi‐color immunostaining panels suitable for the following behavioral studies on native spider silk fibers.

### 3.2 Morphological analysis of rSC, rFB, and rDRG cultures on different substrates supported native spider silk as favorable growth substrate

**FIGURE 3:**
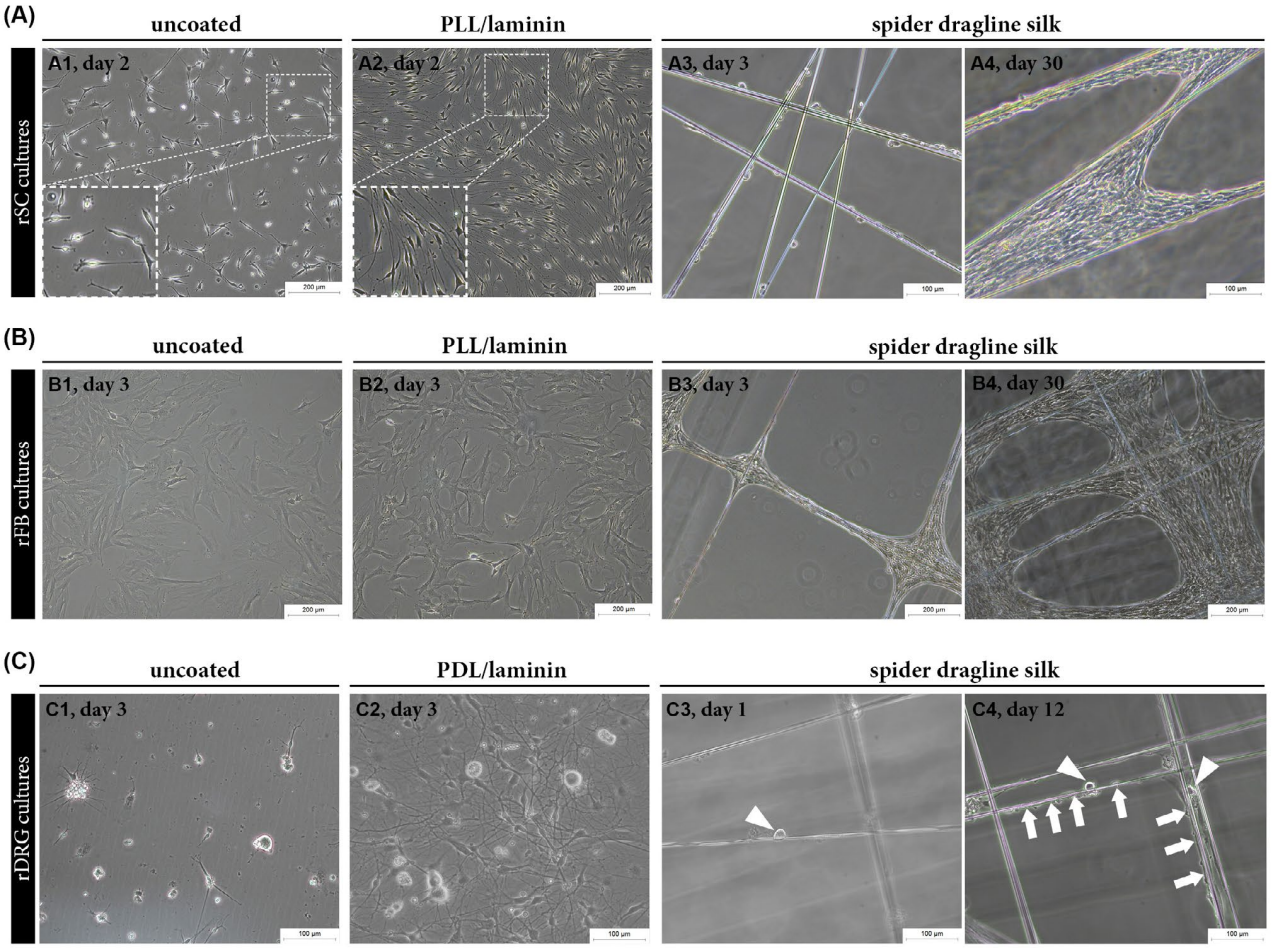
Comparison of the growth of rSCs, rFBs and rDRG neurons on different substrates. A‐C, Representative phase contrast images of rSCs, rFBs, and rDRG neurons cultured on different substrates showing successful adhesion and growth of all cell types on spider dragline silk fibers. A, rSCs cultured on uncoated (A1) and PLL/laminin coated (A2) dishes at day 2 as well as on silk fibers at day 3 (A3) and day 30 (A4). B, rFBs cultured on uncoated (B1) and PLL/laminin (B2) coated dishes at day 4 as well as on silk fibers at day 3 (B3) and day 30 (B4). C, rDRG neurons cultured on uncoated (C1) and PDL/laminin (C2) coated dishes at day 3 as well as on spider silk fibers at day 1 (C3) and day 12 (C4); arrowheads indicate rDRG cell bodies; arrows indicate elongated cells along the silk fibers where rDRGs are present

Next, the growth of rSCs, rFBs, and rDRGs cultured on (a) uncoated dishes, (b) their preferred coating, and (c) dragline spider silk was compared. rSCs grown on uncoated dishes showed impaired spreading and shorter extensions, whereas rSCs possessed the typical spindle‐shaped morphology with long extensions on PLL/laminin coating (Figure 3A1 vs A2). When cultured on spider silk, rSCs successfully adhered and aligned along the fibers (Figure 3A3). After 30 days in culture, rSCs had formed elongated and bundled cell layers (Figure 3A4). No obvious morphological differences of rFB cultures were observed between uncoated and PLL/laminin coated dishes (Figure 3B1 vs B2). Also rFBs accepted silk fibers as growth substrate where they usually accumulated at the fiber crossings (Figure 3B3) and rapidly developed a dense cell layer spreading over the silk mesh (Figure 3B3, B4). rDRG neurons only weakly adhered to uncoated dishes and neurite outgrowth was poor (Figure 3C1) when compared to cultures on PDL/laminin coating, which supported cell attachment and the rapid formation of long axonal processes (Figure 3C2). Of note, the large rDRG neuron cell bodies were able to adhere to the silk fibers (Figure 3C3) and elongated cells, presumably rSCs, were observed next to rDRG neurons with increased culture time (Figure 3C4). However, exact evaluation of axonal out‐growth on silk was impeded by their thin size. These observations indicate that rSCs, rFBs, and rDRG neurons accepted spider silk as growth substrate without the need of any modification, while coating of culture dishes was necessary to allow the proper adhesion of rSC and rDRG neurons.

### 3.3 Confocal imaging of rSC, rFB, and rDRG neurons cultures grown on spider silk confirmed cell‐typical behavior and marker expression

**FIGURE 4:**
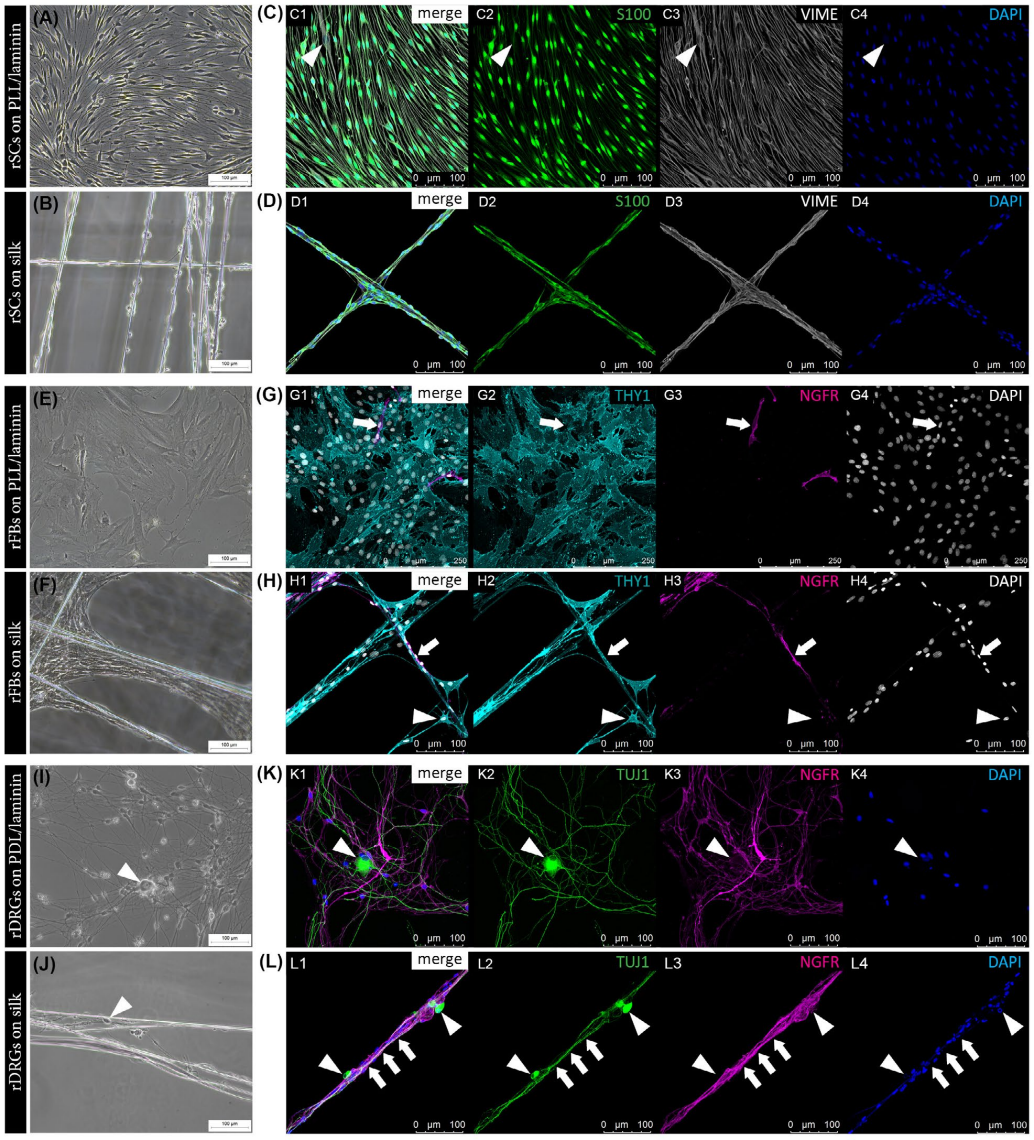
Phase contrast *versus* confocal imaging of rSC, rFB, and rDRG cultures on different substrates. Representative phase contrast images of rSC cultures on PLL/laminin (A) and on silk fibers (B). IF images of rSC cultures on PLL/laminin (C) and on spider silk (D) with merged channels (C1, D1) and single channels for S100 in green (C2, D2), VIME in grey (C3, D3) and DAPI in blue (C4, D4) showing a similar alignment and elongated morphology; arrowheads indicate a S100^−^/VIME^+^ rFB. Representative phase contrast images of rFB cultures on PLL/laminin (E) and on silk fibers (F). IF images of rFB cultures on PLL/laminin (G) and on spider silk (H) with merged channels (G1, H1) and single channels for NGFR in magenta (G2, H2), THY1 in cyan (G3, H3) and DAPI in grey (G4, H4) illustrating an increased elongation of rFBs on silk; arrows indicate a NGFR^+^/THY1^−^ rSC, arrowheads indicate a NGFR^−^/THY1^+^ rFB spanning across silk fibers. Representative phase contrast images of rDRG cultures on PDL/laminin (I) and on silk fibers (J). IF images for rDRG cultures on PDL/laminin (K) and on spider silk (L) with merged channels (K1, L1) and single channels for TUJ1 (beta‐3‐tubulin) in green (K2, L2), NGFR in magenta (K3, L3), and DAPI in blue (K4, L4) revealing bundled TUJ1^+^ axonal processes surrounded by NGFR^+^ rSCs (arrows); arrowheads indicate rDRG neuron cell bodies

For a more detailed comparison of the morphology and behavior of rSCs, rFBs, and rDRG neurons between spider silk and controls, the established immunofluorescence staining panels (see Figure 2) were used. Phase contrast images suggested that rSCs elongated and aligned on PLL/laminin and silk fibers (Figure 4A,B). Confocal imaging enabled a detailed visualization of single cells and confirmed that rSCs had a similar spindle‐shaped morphology with long processes on both substrates (Figure 4C,D). The flat and broad appearance of rFBs observed on PLL/coating changed to a more elongated form on the silk fibers, but the high cell density on the latter impeded proper morphological characterization via phase contrast microscopy (Figure 4E,F). The confocal images illustrated the spread cell morphology rFBs on PLL/laminin, while spider silk indeed encouraged rFB elongation, and also the spanning of cell bodies across the fibers (Figure 4G,H). When rDRG neurons were monitored with phase contrast microscopy, the fine and thin axonal processes developed on PDL/laminin could be hardly assessed on silk fibers (Figure 4I vs J). The confocal images confirmed a widely spread axonal network on PDL/laminin with co‐localized rSCs (Figure 4K) but, notably, revealed that rDRG neurons extended sustained axonal processes along the silk fibers, which appeared bundled and aligned with rSCs (Figure 4L). Hence, the established immunostaining panels combined with confocal microscope analysis facilitated a detailed and cell type‐specific assessment of the morphological features of rSC, rFB, and rDRG cultures on silk. The results illustrate that the respective cellular characteristics, which usually only develop on a specific coating substrate, are preserved on untreated, native spider silk fibers.

### 3.4 Quantification of rSC and rFB culture purity and proliferation in response to spider silk fibers

**FIGURE 5:**
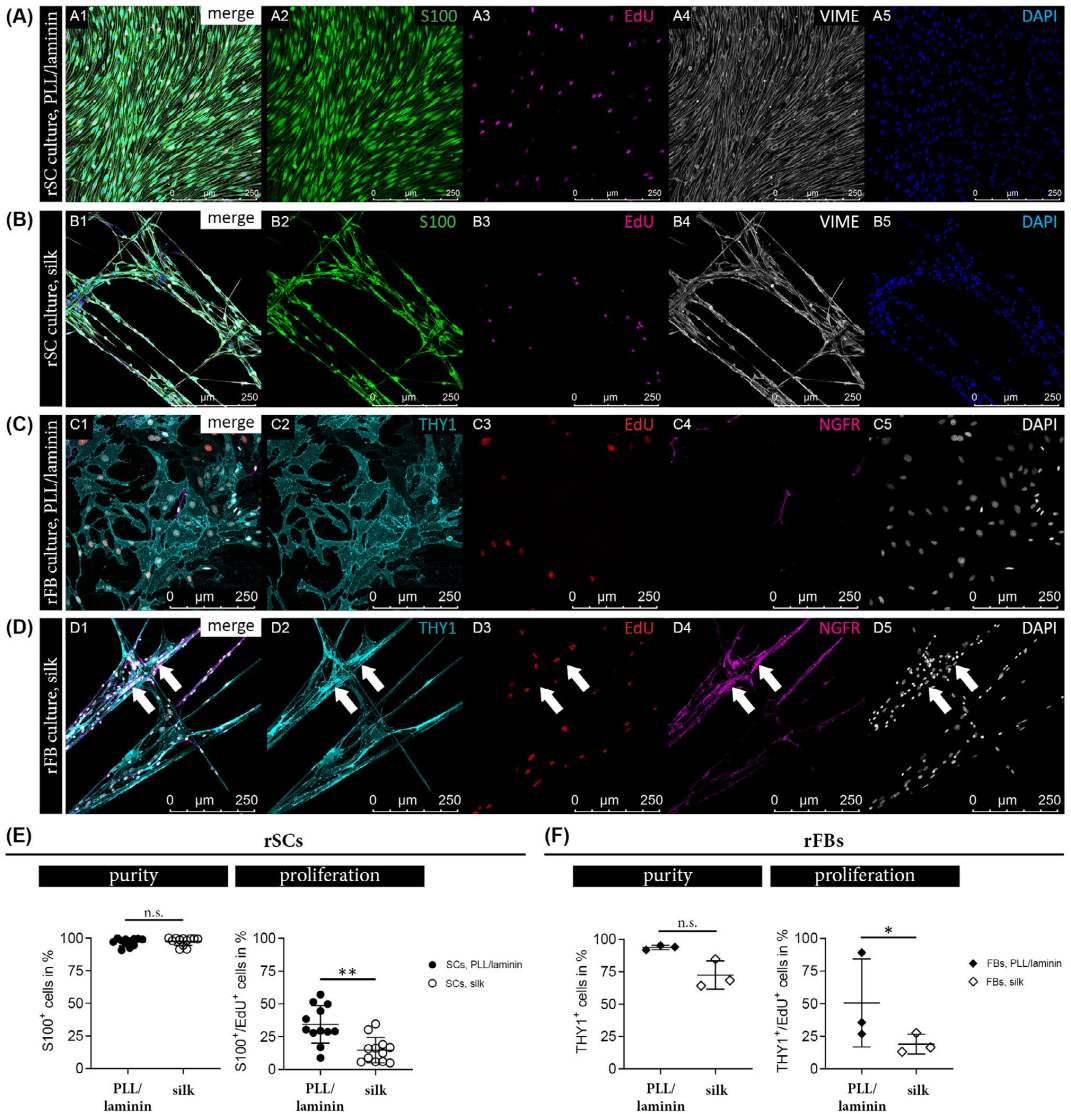
Comparison of rSC and rFB purity and proliferation on different substrates. IF stainings of rSCs cultured on PLL/laminin (A) and silk fibers (B) with merged channels (A1, B1) and single channels for S100 in green (A2, B2), EdU proliferation marker in magenta (A3, B3), VIME in grey (A4, B4) and DAPI in blue (A5, B5). IF stainings of rFBs cultured on PLL/laminin (C) and silk fibers (D) with merged channels (C1, D1) and single channels for THY1 in cyan (C2, D2), EdU proliferation marker in red (C3, D3), NGFR in magenta (C4, D4) and DAPI in grey (C5, D5); arrows indicate accumulated NGFR^+^ rSCs on top of THY1^+^ rFBs. E, Purity and proliferation of rSCs cultures on PLL/laminin and silk. Diagrams depict the percentage of S100^+^ SCs (left) and the percentage of S100^+^/EdU^+^ SCs (right) in each condition ± SD (n = 12); n.s. (not significant), **P‐value < .01. F, Purity and proliferation of rFBs cultures on PLL/laminin and silk. Diagrams depict the percentage of THY1^+^ FBs (left) and the percentage of THY1^+^/EdU^+^ FBs (right) in each condition ± SD (n = 3); n.s. (not significant), *P‐value < .05

Further, we aimed to compare the purity and proliferation rate of rSC and rFB grown on PLL/laminin and silk fibers. After about 20 days of culture, EdU detection was performed to visualize DNA synthesizing cells followed by respective immunostaining panels established for rSC (Figure 5A,B) and rFB cultures (Figure 5C,D). Multi‐color confocal images enabled the quantification of S100^+^/VIME^+^ rSCs and S100^+^/EdU^+^/VIME^+^ proliferating rSCs in rSC cultures as well as THY1^+^/NGFR^−^ rFBs and THY1^+^/EdU^+^/NGFR^−^ proliferating rFBs in rFB cultures. A mean rSC culture purity of about 95% was determined on both substrates, while proliferation of rSCs was significantly decreased on silk (Figure 5E). rFB cultures on PLL/laminin possessed a purity >90% but, interestingly, their purity on silk tended to decrease due to emerging accumulations of rSCs (Figure 5F). Similar to rSC cultures, the rFB proliferation rate was significantly reduced when cultured on silk fibers (Figure 5F). These results showed that rSCs and rFBs proliferated on the silk fibers but that the proliferation rate was reduced when compared to cultures on PLL/laminin substrate.

### 3.5 Quantification of the migratory potential of rSCs and rFBs on spider silk fibers

**FIGURE 6:**
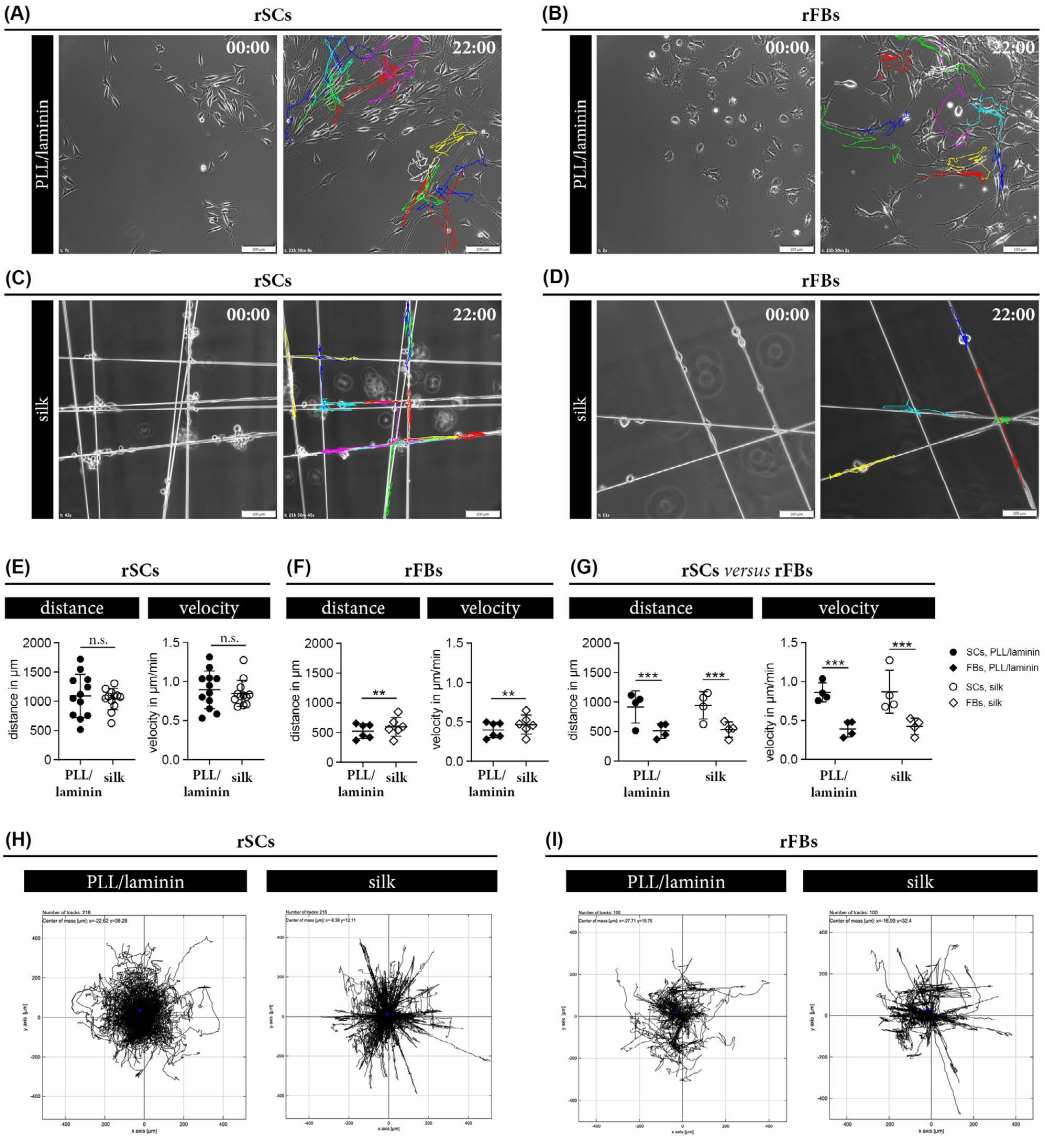
Comparison of the migratory behavior of rSC and rFB on different substrates. A‐D, Representative phase contrast images of rSCs cultured on PLL/laminin (A) and silk (C) as well as rFBs cultured on PLL/laminin (B) and silk (D) at the start (00:00) and end (22:00) of live cell imaging. Each colored trajectory illustrates the tracking result for one cell after 22 hours. (E, F) Diagrams depict the migration distance in µm and the velocity in µm/min ± SD of rSCs (n = 12) (E) and rFBs (n = 7) (F) on PLL/laminin and silk substrates; n.s. (not significant), **P‐value < .01. G, Comparison of the migratory potential between rSCs and rFBs derived from the same donor (n = 4) on the two different substrates showed a significantly higher covered migration distance and the velocity of rSCs; ***P‐value < .001. (H, I)Migration plots of rSCs (H) and rFBs (I) cultured on PLL/laminin and silk; the trajectory of each cell starts in the middle and depicts the migration direction in µm

To provide insight into the migratory behavior of rSCs and rFBs on PLL/laminin and silk fiber substrates, we performed live cell imaging of freshly seeded cultures by taking one picture per 10 minutes for 22 hours. Individual cells were then manually tracked throughout the 132 pictures to determine the mobility of cells on the two different substrates. The results were visualized in form of colored trajectories for rSCs and rFBs on PLL/laminin (Figure 6A,B) and on silk fibers (Figure 6C,D). The live cell imaging videos with the trajectories of rSCs and rFBs cultured on silk are available in Videos S1 and S2, respectively, and visualized a rather uncoordinated movement of rSCs and rFBs. Based on the trajectory data, the mean velocity (µm/min) as well as the mean migration distance (in µm) within 22 hours of observation were calculated. The covered distance by the rSCs was 1,100 µm and their velocity was 0.9 µm/min, and both were similar on PLL/laminin and silk (Figure 6E). rFBs reached a migration distance of 500 µm and a velocity of 0.4 µm/min and those values were marginally but significantly increased to 600 µm and 0.5 µm/min on the silk fibers (Figure 6F). By comparing the migratory potential between rSCs and rFBs derived from the same donors, we confirmed that both the covered distance and the velocity of rSCs were about 50% higher than those of rFBs independent of the substrate (Figure 6G). The migration plots visualized the migration behavior of rSCs (Figure 6H) and rFBs (Figure 6I) on silk and PLL/laminin. In summary, the data revealed that the migratory potential of rSCs is about twice as high than that of rFBs and that silk fibers allowed rSCs to cover a migration distance of 1.1 mm within 22 hours, which is in line with the average nerve regeneration rate of 1 mm per day.^21^

**FIGURE 7:**
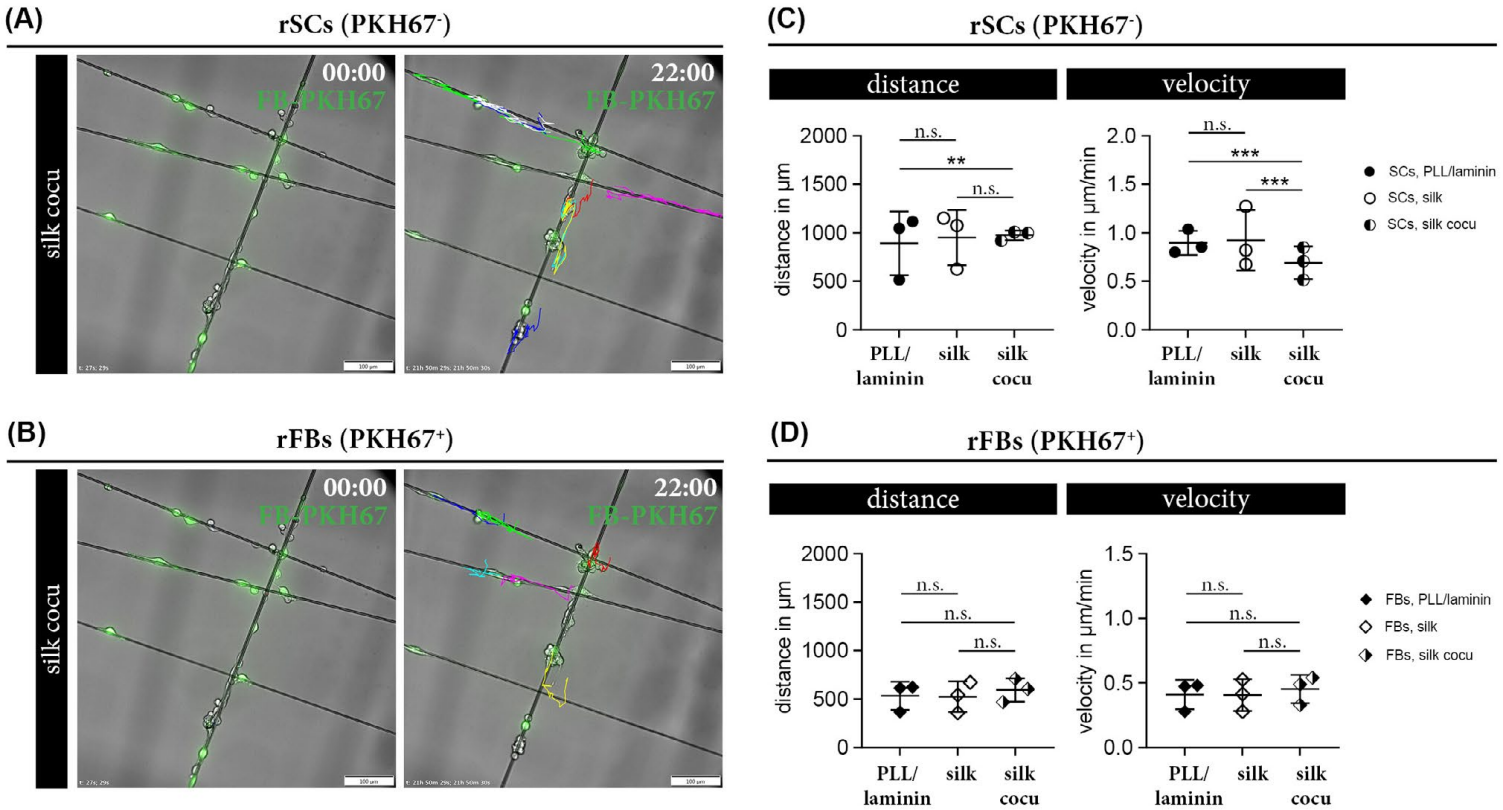
Comparison of the migratory behavior of rSC and rFB co‐cultures. (A, B) Representative overlay image of phase contrast and corresponding green fluorescent channel visualizes PKH67^−^ rSCs (A) and PKH67^+^ rFBs (B) in co‐cultures on silk at the start (00:00) and end (22:00) of live cell imaging. Colored trajectories illustrate the tracking results for PKH67^−^ rSCs (A) and PKH67^+^ rFBs (B) after 22 hours. (C, D)Diagrams depict the covered distance in µm and the velocity in µm/min ± SD of rSCs (n = 3) (C) and rFBs (n = 3) (D) cultured on PLL/laminin, silk, and silk co‐cultures; n.s. (not significant), **P‐value < .01, ***P‐value < .001

We further analyzed the impact of a rFB population on the migratory behavior of rSCs cultured on silk. Discrimination of rSCs and rFBs was achieved by labeling rFBs with the green fluorescent cell membrane dye PKH67 prior to the co‐culture. PKH stably anchors fluorophores with aliphatic tails in the lipid regions of cell membranes, allowing bright labeling of cells over an extended period of time. Live cell imaging of these co‐cultures was performed for 22 hours. PKH67^−^ rSCs (Figure 7A) and PKH67^+^ rFBs (Figure 7B) were manually tracked and results were compared to respective pure cultures on PLL/laminin and on silk. While the presence of rFBs hardly affected the covered migration distance of rSCs, they significantly decreased the velocity of rSCs when compared to pure rSC cultures (Figure 7C). In turn, the presence of rSCs had no effect on the migratory behavior of rFBs (Figure 7D).

## 4 DISCUSSION

The enrichment of hollow nerve conduits with internal guiding structures or luminal fillers is suggested to overcome their poor performance in long‐distance nerve defects.^35^, ^80^, ^81^ Intriguingly, the use of native spider silk fibers applied as internal guiding filaments in nerve conduits achieved a regenerative outcome similar to autologous nerve grafts in large‐distance nerve defects of rats and sheep.^47^, ^59^ However, native spider silk remains a natural material with associated limitations for clinical use. To replace native spider silk with a recombinant analog, it is important to understand how native spider silk supports the regeneration of nerves and which modifications of recombinant silk could be useful to enhance its performance. Hence, qualitative and quantitative parameters are needed to identify and evaluate the recombinant silk candidates suitable for future in vivo studies. To address that need, this study aimed to deduce the regenerative effects of native spider silk by analyzing the behavior and interaction of three major cell types involved in nerve repair (a) primary SCs, (b) nerve‐associated FBs and (c) DRG‐derived sensory neurons in response to native spider silk fibers in vitro.

Our results confirmed that coating with laminin, an ECM protein, and positively charged amino acid polymers (PLL and PDL) is necessary for proper adhesion and outgrowth of rSCs and rDRG neurons on plastic culture dishes. Remarkably, primary rSCs, rFBs as well as rDRG neurons attached to native spider silk fibers without any additional treatment. Furthermore, all cell types cultured on the native silk fibers possessed their characteristic morphological features: rSCs aligned in a bipolar fashion, rFBs spread across the fibers, and rDRG neurons formed long axonal processes. In order to facilitate a detailed assessment of cell‐specific behavior on the silk, we established multi‐color immunofluorescence staining panels for rSCs, rFBs, and rDRG neurons. Confocal image analysis illustrated that rSCs aligned along the silk fibers and formed longitudinal cell strands resembling the regeneration tracks known as Bungner bands. Hence, the properties of native spider silk fibers likely mimic the basal lamina tubes repair SCs use to orientate and align after nerve injury. Repair SCs release neurotrophic factors and offer the preferred substrate for axons as they express neuritogenic cues such as neural adhesion molecules and basal lamina proteins.^11^, ^82^, ^83^ Accordingly, rDRG neurons extended their axonal processes in close association with longitudinally aligned rSCs on the silk. The formation of Bungner bands along native spider silk fibers could be responsible for the effective regeneration through nerve conduits in vivo^48^, ^59^ by providing essential topographical guidance structures for a directed and fast re‐growth of axons. We recommend to use super‐resolution microscopy to further analyze the Bungner band‐like structures on silk fibers in detail and gain information about axon properties such as the number of neurites, diameter, and growth cones, as well as the produced basal lamina proteins by SCs. The native spider silk also favored elongation and spreading of nerve‐associated rFBs across the silk fibers, which is in line with the fast development of dense cell layers on spider silk meshes reported previously for NIH/3T3 FBs.^57^ While the formation of cell layers across silk fibers could be beneficial for other tissue engineering applications, for example, bladder reconstructions,^67^ excessive FB spreading and distribution within internal guiding filaments of nerve conduits might block the regeneration tracks and impede nerve repair.

Internal guiding filaments for nerve conduits should further allow cell proliferation to populate the conduit with cells that provide a growth supportive milieu. However, a low proliferation rate of nerve resident FBs is associated with a reduced fibrosis risk, while a high proliferation rate of SCs is associated with a beneficial regenerative effect. In order to determine the proliferation status of rSCs and rFBs in response to native silk fibers, EdU detection was implemented in the established staining panels for the quantitative evaluation of DNA synthesizing cells. Both rSCs and rFBs cultured on silk proliferated but their proliferation rate was reduced when compared to cultures on PLL/laminin coating, where cells undergo regular passaging. This could be caused by the silk itself or by a contact mediated inhibition of proliferation induced by the close proximity of cells cultured on silk^84^ that, however, more adequately reflect the in vivo situation. Although a decreased proliferation of SCs could be considered disadvantageous, an in vivo study demonstrated that the inhibition of SC proliferation during nerve injury did not markedly affect the regenerative outcome.^85^ It was concluded that rather the maintenance of repair‐specific SC functions than SC proliferation is essential for nerve regeneration.^15^ Hence, further studies that investigate whether the repair‐specific functional spectrum of rSCs is preserved on silk are highly desirable. In addition to proliferation, also the ECM protein expression profile of FBs defines their activation status. To exclude an aberrant deposition of ECM by nerve‐associated FBs in response to (recombinant) silk fibers, we suggest to additionally define their repertoire of expressed ECM proteins in future studies.

Time is a critical factor for functional recovery of long nerve defects as the re‐growing axons need to reach the target organ before chronic denervation leads to functional loss.^16^, ^86^, ^87^ The successful migration of SCs along the internal guiding filaments is, thus, essential to direct the re‐growing axons through the nerve conduit. Herein, live cell imaging was used to provide insight into the migratory behavior of rSCs and rFBs on the native spider silk fibers for 22 hours. Our results demonstrated that the inherent migratory potential of rSCs, measured by the migration distance and velocity, is more than twice as high than that of rFBs. Most remarkable was the mean rSC migration distance of 1.1 mm within 22 hours of culture on the silk fibers, which reflects the reported axon growth rate of about 1‐3 mm per day.^21^, ^88^ This finding underlines that native spider silk fibers allow a physiological relevant migration of rSCs, an important feature to precede and guide axons across the nerve defect. However, the migratory behavior of rSCs on native spider silk also included uncoordinated movements that reduced their effective migration distance. Increasing the directionality of SC migration on silk fibers is a promising strategy to improve the guidance of axons across large‐distance nerve defects. Moreover, we showed that the presence of rFBs significantly decreased the velocity of rSCs on silk fibers. This result supported existing SC‐FB interactions via cell adhesion molecules,^71^ but argues against a benefit of nerve‐associated FBs seeded in silk fiber enriched nerve conduits as they will counteract the remarkable migration potential of SCs.

This study showed that the regenerative effect of native spider silk during nerve repair is presumably due to its excellent adhesive properties that also facilitate the migratory capacity of SCs and the formation of Bungner band‐like structures. Despite these compelling results, we also found that SCs cultured on native spider silk lacked migration directionality. These findings represent an essential first step to adjust the properties of recombinant silk fibers for nerve repair. The variety of recombinant spider silk proteins is constantly improving and up‐and‐coming to be exploited in form of luminal fillers and internal guiding filaments for nerve conduits. Importantly, the production process of recombinant spider silk proteins enables to optimize the properties of the generated scaffold by genetic modifications or biofunctionalization that can selectively affect cell adhesion, differentiation, and function.^60^ It is, thus, important to identify the molecular basis behind the regenerative effect of native spider silk to tailor the features of recombinant silk to the specific needs for nerve repair. However, the material properties underlying the excellent interactions of cells with native spider silk are poorly understood but likely involve a combination of factors. A recent study showed that the attachment of rat SCs to different kinds of native spider silk fibers is independent of their diameter, but that SC elongation and distribution along the fibers was associated with a high content of β‐sheets within the silks secondary protein structure.^89^ Cell attachment and function can also be influenced by charged functional groups.^90^, ^91^ While low cell adhesion was observed to *Bombyx mori* silk fibroin that possess a high density of negative charges, we and others demonstrated good cell attachment and proliferation to native spider silk that is assembled of proteins with positive charges.^48^, ^56^, ^57^, ^89^, ^92^ Proof of principle was provided by the different adhesion of cells to silk films composed of positively or negatively charged variants of the same recombinant spider silk protein eADF4(C16).^93^ Hence, the β‐sheet content and charge are important parameters of native spider silk fibers that should be preserved in recombinant silk, whereas distinct modifications could overcome the limitation of native silk.

As we found that a FBs significantly reduce the velocity of SCs on silk fibers, desirable modifications to recombinant silk proteins could contain selective binding signals that either increase the affinity of SCs or reduce the binding of FBs to the silk fiber.^94^ Ways to modify recombinant silk proteins include physical adsorption, chemical modifications of amino acid side chains, and genetic engineering.^95^ Several studies supported that recombinant spider silk‐based matrices modified with cell binding motifs derived from ECM proteins can promote the adhesion of various cell types,^95^, ^96^, ^97^ and that the IKVAV motif had a significant effect on both adhesion and survival of SCs.^97^ Incorporating such peptide sequences into longitudinally aligned recombinant silk fibers could promote the early adhesion and Bungner band formation of SCs to hasten nerve regeneration. Our results also showed that the migratory behavior of SCs on the native spider silk is excellent but lacks directionality. As the neurite outgrowth from the proximal nerve stump follows the lead of SCs,^98^ improving the directionality of SC migration along the silk fibers will likely result in an increased guidance of axons through the conduit. For example, filaments composed of recombinant silk could be aligned inside the lumen of nerve conduits that contain a gradient of bioactive molecules promoting SC migration, such as GDNF‐loaded silk microspheres incorporated in the conduit wall.^99^ Alternatively, nerve conduits that comprise a combination of (a) silk fibers as guiding structure and (b) hydrogels as source of bioactive molecules might be ideal to support the requirements for nerve regeneration. While recombinant silk fibers serve as topographical guidance, the hydrogel can include a variety of nerve repair promoting strategies such as the controlled release of neurotrophic factors, electro‐conducting polymers, or cues influencing cell adhesion and function, reviewed in.^100^ Moreover, hydrogels allow to incorporate factors that block inhibitors of neural regeneration, for example chondroitinase ABC that counteract fibrotic scar formation by degrading chondroitin sulfate proteoglycans.^101^ With respect to long‐distance nerve defects, nerve conduits also need to be enriched with cells to replace the lost tissue and as a source for neurotrophic support. Transplanted autologous SCs or easier accessible mesenchymal stem cells are known to increase nerve regeneration.^102^, ^103^, ^104^, ^105^ The efficient delivery and survival of transplanted cells could be improved by their encapsulation in degrading hydrogels that allow the exchange of soluble molecules and nutrients.^106^

Indeed, the fabrication of a multifunctional nerve conduit to treat long‐distance nerve defects in patients will demand a combinatorial approach comprising the kind of conduit, internal guiding structures, luminal fillers, functionalization, and cells to achieve clinical relevant results. Using the regenerative features of native spider silk fibers as baseline, this study provides a first step to implement recombinant silk fibers as internal guiding filaments for nerve conduits. We defined reference parameters recombinant silk fibers should meet for nerve repair and contribute suitable assays to compare their performance in vitro and identify the most promising candidates eligible for future in vivo studies.

## 5 CONCLUSION

Herein, we established read‐outs for a refined and detailed behavioral analysis of primary rSCs, nerve‐associated rFBs, and rDRG neurons cultured on native dragline silk. Our results demonstrate that native spider silk fibers possess excellent adhesive properties allowing cell alignment, proliferation, and migration without the need of any modification. Moreover, rSCs form sustained bundled structures along the silk fibers that were populated with outgrowing axonal processes of rDRG neurons. Thus, native spider silk fibers provide a framework that substitutes basal lamina tubes as topographical guidance structure by supporting the intrinsic nature of repair SCs to form Bungner bands for re‐growing axons. Native spider silk also allowed an eminent migration of rSCs but their directionality was poor. We suggest to enhance the regeneration of axons through nerve conduits by increasing the directionality of SC migration using recombinant spider silk fibers in combination with functionalized hydrogels.

## CONFLICT OF INTEREST

The authors declare no conflict of interest.

## AUTHOR CONTRIBUTIONS

Conceptualization: T. Weiss, F. Millesi, and C. Radtke; Methodology & Investigation: F. Millesi, T. Weiss, A. Mann, and M. Haertinger; Material: L. Semmler, F. Millesi, and P. Supper; Software: F. Millesi; Visualization: T. Weiss and F. Millesi; Formal Analysis: F. Millesi, A. Naghilou, T. Weiss, and D. Pils; Writing & Draft Preparation: T. Weiss and F. Millesi; Writing‐review & Editing: T. Weiss, F. Millesi, M. Haertinger, A. Mann, P. Supper, L. Semmler, D. Pils, A. Naghilou, and C. Radtke; Project Administration: T. Weiss; Supervision & Funding Acquisition: C. Radtke.

## Supporting information

Table S1

Video S1

Video S2

## References

[1] Cunha C , Panseri S , Antonini S . Emerging nanotechnology approaches in tissue engineering for peripheral nerve regeneration. Nanomedicine. 2011;7(1):50‐59. 10.1016/j.nano.2010.07.004 20692373

[2] Gaudin R , Knipfer C , Henningsen A , Smeets R , Heiland M , Hadlock T . Approaches to peripheral nerve repair: generations of biomaterial conduits yielding to replacing autologous nerve grafts in craniomaxillofacial surgery. Biomed Res Int. 2016;2016:1–18. 10.1155/2016/3856262. PMC4983313 27556032

[3] Lundborg G . A 25‐year perspective of peripheral nerve surgery: evolving neuroscientific concepts and clinical significance. J Hand Surg Am. 2000;25(3):391‐414. 10.1053/jhsu.2000.4165 10811744

[4] Millesi H . Bridging defects: autologous nerve grafts. Acta Neurochir Suppl. 2007;100:37‐38. 10.1007/978-3-211-72958-8_8 17985542

[5] Taylor CA , Braza D , Rice JB , Dillingham T . The incidence of peripheral nerve injury in extremity trauma. Am J Phys Med Rehabil. 2008;87(5):381‐385. 10.1097/PHM.0b013e31815e6370 18334923

[6] Ciaramitaro P , Mondelli M , Logullo F , et al. Traumatic peripheral nerve injuries: epidemiological findings, neuropathic pain and quality of life in 158 patients. J Peripher Nerv Syst. 2010;15(2):120‐127. 10.1111/j.1529-8027.2010.00260.x 20626775

[7] Seddon HJ . Three types of nerve injury. Brain. 1943;66(4):237‐288. 10.1093/brain/66.4.237

[8] Arthur‐Farraj P , Latouche M , Wilton D , et al. c‐Jun reprograms Schwann cells of injured nerves to generate a repair cell essential for regeneration. Neuron. 2012;75(4):633‐647. 10.1016/j.neuron.2012.06.021 22920255 PMC3657176

[9] Jang SY , Shin YK , Park SY , et al. Autophagic myelin destruction by schwann cells during wallerian degeneration and segmental demyelination. Glia. 2016;64(5):730‐742. 10.1002/glia.22957 26712109

[10] Gomez‐Sanchez JA , Carty L , Iruarrizaga‐Lejarreta M , et al. Schwann cell autophagy, myelinophagy, initiates myelin clearance from injured nerves. J Cell Biol. 2015;210(1):153‐168. 10.1083/jcb.201503019 26150392 PMC4494002

[11] Jessen KR , Mirsky R . The repair Schwann cell and its function in regenerating nerves. J Physiol. 2016;594(13):3521‐3531. 10.1113/JP270874 26864683 PMC4929314

[12] Weiss T , Taschner‐Mandl S , Bileck A , et al. Proteomics and transcriptomics of peripheral nerve tissue and cells unravel new aspects of the human Schwann cell repair phenotype. Glia. 2016;64(12):2133‐2153. 10.1002/glia.23045 27545331

[13] Stoll G , Muller HW . Nerve injury, axonal degeneration and neural regeneration: basic insights. Brain Pathol. 1999;9(2):313‐325. 10.1111/j.1750-3639.1999.tb00229.x 10219748 PMC8098499

[14] Hoke A , Brushart T . Introduction to special issue: Challenges and opportunities for regeneration in the peripheral nervous system. Exp Neurol. 2010;223(1):1‐4. 10.1016/j.expneurol.2009.12.001 20004660 PMC3071992

[15] Jessen KR , Mirsky R . The success and failure of the Schwann cell response to nerve injury. Front Cell Neurosci. 2019;13:33. 10.3389/fncel.2019.00033 30804758 PMC6378273

[16] Sulaiman OA , Gordon T . Role of chronic Schwann cell denervation in poor functional recovery after nerve injuries and experimental strategies to combat it. Neurosurgery. 2009;65(4 Suppl):A105‐A114. 10.1227/01.neu.0000358537.30354.63 19927054

[17] Ray WZ , Mackinnon SE . Management of nerve gaps: autografts, allografts, nerve transfers, and end‐to‐side neurorrhaphy. Exp Neurol. 2010;223(1):77‐85. 10.1016/j.expneurol.2009.03.031 19348799 PMC2849924

[18] Millesi H , Schmidhammer R . End‐to‐side coaptation–controversial research issue or important tool in human patients. Acta Neurochir Suppl. 2007;100:103‐106. 17985556 10.1007/978-3-211-72958-8_22

[19] Johnson EO , Zoubos AB , Soucacos PN . Regeneration and repair of peripheral nerves. Injury. 2005;36(Suppl 4):S24‐S29. 10.1016/j.injury.2005.10.012 16288757

[20] Millesi H , Meissl G , Berger A . The interfascicular nerve‐grafting of the median and ulnar nerves. J Bone Joint Surg Am. 1972;54(4):727‐750. 4560075

[21] Grinsell D , Keating CP . Peripheral nerve reconstruction after injury: a review of clinical and experimental therapies. Biomed Res Int. 2014;2014:1–13. 10.1155/2014/698256 PMC4167952 25276813

[22] Kanno H , Pearse DD , Ozawa H , Itoi E , Bunge MB . Schwann cell transplantation for spinal cord injury repair: its significant therapeutic potential and prospectus. Rev Neurosci. 2015;26(2):121‐128. 10.1515/revneuro-2014-0068 25581750

[23] Arslantunali D , Dursun T , Yucel D , Hasirci N , Hasirci V . Peripheral nerve conduits: technology update. Med Devices (Auckl). 2014;7:405‐424. 10.2147/mder.s59124 25489251 PMC4257109

[24] Lovati AB , D’Arrigo D , Odella S , Tos P , Geuna S , Raimondo S . Nerve repair using decellularized nerve grafts in rat models. A review of the literature. Frontiers in Cellular Neuroscience. 2018;12(427). 10.3389/fncel.2018.00427 PMC6254089 30510503

[25] Muheremu A , Ao Q . Past, present, and future of nerve conduits in the treatment of peripheral nerve injury. Biomed Res Int. 2015;2015:1–6. 10.1155/2015/237507 PMC4600484 26491662

[26] Kehoe S , Zhang XF , Boyd D . FDA approved guidance conduits and wraps for peripheral nerve injury: a review of materials and efficacy. Injury. 2012;43(5):553‐572. 10.1016/j.injury.2010.12.030 21269624

[27] Vijayavenkataraman S . Nerve guide conduits for peripheral nerve injury repair: a review on design, materials and fabrication methods. Acta Biomater. 2020;106:54‐69. 10.1016/j.actbio.2020.02.003 32044456

[28] Jiang X , Lim SH , Mao HQ , Chew SY . Current applications and future perspectives of artificial nerve conduits. Exp Neurol. 2010;223(1):86‐101. 10.1016/j.expneurol.2009.09.009 19769967

[29] Schlosshauer B , Dreesmann L , Schaller HE , Sinis N . Synthetic nerve guide implants in humans: a comprehensive survey. Neurosurgery. 2006;59(4):740‐748. 10.1227/01.NEU.0000235197.36789.42 17038939

[30] Moore AM , Kasukurthi R , Magill CK , Farhadi HF , Borschel GH , Mackinnon SE . Limitations of Conduits in Peripheral Nerve Repairs. Hand (N Y). 2009;4(2):180‐186. 10.1007/s11552-008-9158-3 19137378 PMC2686795

[31] Kaplan HM , Mishra P , Kohn J . The overwhelming use of rat models in nerve regeneration research may compromise designs of nerve guidance conduits for humans. J Mater Sci Mater Med. 2015;26(8):226. 10.1007/s10856-015-5558-4 26296419 PMC4545171

[32] Moore AM , MacEwan M , Santosa KB , et al. Acellular nerve allografts in peripheral nerve regeneration: a comparative study. Muscle Nerve. 2011;44(2):221‐234. 21660979 10.1002/mus.22033 PMC3136642

[33] Carvalho CR , Oliveira JM , Reis RL . Modern trends for peripheral nerve repair and regeneration: beyond the hollow nerve guidance conduit. Front Bioeng Biotechnol. 2019;7:337. 10.3389/fbioe.2019.00337 31824934 PMC6882937

[34] Daroff RB , Jankovic J , Mazziotta JC , Pomeroy SL . Bradley's neurology in clinical practice, 7th edn. New York, NY: Elsevier; 2016.

[35] Lundborg G , Dahlin L , Dohi D , Kanje M , Terada N . A new type of “bioartificial” nerve graft for bridging extended defects in nerves. J Hand Surg: Br Eur Vol. 1997;22(3):299‐303. 10.1016/S0266-7681(97)80390-7 9222905

[36] Reimers K , Liebsch C , Radtke C , Kuhbier JW , Vogt PM . Silks as scaffolds for skin reconstruction. Biotechnol Bioeng. 2015;112(11):2201‐2205. 10.1002/bit.25654 25995140

[37] Seal BL , Otero TC , Panitch A . Polymeric biomaterials for tissue and organ regeneration. Mater Sci Eng R Rep. 2001;34(4):147‐230. 10.1016/S0927-796X(01)00035-3

[38] Pabari A , Yang SY , Mosahebi A , Seifalian AM . Recent advances in artificial nerve conduit design: strategies for the delivery of luminal fillers. J Control Release. 2011;156(1):2‐10. 10.1016/j.jconrel.2011.07.001 21763371

[39] Lally KP , Cheu HW , Vazquez WD . Prosthetic diaphragm reconstruction in the growing animal. J Pediatr Surg. 1993;28(1):45‐47. 8429470 10.1016/s0022-3468(05)80352-5

[40] Schafer‐Nolte F , Hennecke K , Reimers K , et al. Biomechanics and biocompatibility of woven spider silk meshes during remodeling in a rodent fascia replacement model. Ann Surg. 2014;259(4):781‐792. 10.1097/SLA.0b013e3182917677 23873006

[41] Brown CN , Finch JG . Which mesh for hernia repair? Ann R Coll Surg Engl. 2010;92(4):272‐278. 10.1308/003588410X12664192076296 20501011 PMC3025220

[42] Sosa I , Reyes O , Kuffler DP . Immunosuppressants: neuroprotection and promoting neurological recovery following peripheral nerve and spinal cord lesions. Exp Neurol. 2005;195(1):7‐15. 10.1016/j.expneurol.2005.04.016 15935348

[43] Anderson JM , Rodriguez A , Chang DT . Foreign body reaction to biomaterials. Semin Immunol. 2008;20(2):86‐100. 10.1016/j.smim.2007.11.004 18162407 PMC2327202

[44] Herrera J , Henke CA , Bitterman PB . Extracellular matrix as a driver of progressive fibrosis. J Clin Invest. 2018;128(1):45‐53. 10.1172/JCI93557 29293088 PMC5749528

[45] Evans GRD , Brandt K , Katz S , et al. Bioactive poly(L‐lactic acid) conduits seeded with Schwann cells for peripheral nerve regeneration. Biomaterials. 2002;23(3):841‐848. 10.1016/S0142-9612(01)00190-9 11774850

[46] Aigner TB , DeSimone E , Scheibel T . Biomedical applications of recombinant silk‐based materials. Adv Mater. 2018;30(19):e1704636. 10.1002/adma.201704636 29436028

[47] Allmeling C , Jokuszies A , Reimers K , et al. Spider silk fibers in artificial nerve constructs promote peripheral nerve regeneration. Cell Prolif. 2008;41(3):408‐420. 10.1111/j.1365-2184.2008.00534.x 18384388 PMC6496660

[48] Allmeling C , Jokuszies A , Reimers K , Kall S , Vogt PM . Use of spider silk fibers as an innovative material in a biocompatible artificial nerve conduit. J Cell Mol Med. 2006;10(3):770‐777. 10.1111/j.1582-4934.2006.tb00436.x 16989736 PMC3933158

[49] Radtke C , Allmeling C , Waldmann K‐H , et al. Spider silk constructs enhance axonal regeneration and remyelination in long nerve defects in sheep. PLoS One. 2011;6(2):e16990. 10.1371/journal.pone.0016990 21364921 PMC3045382

[50] Tokareva O , Jacobsen M , Buehler M , Wong J , Kaplan DL . Structure‐function‐property‐design interplay in biopolymers: spider silk. Acta Biomater. 2014;10(4):1612‐1626. 10.1016/j.actbio.2013.08.020 23962644 PMC3926901

[51] Cao TT , Zhang YQ . Processing and characterization of silk sericin from Bombyx mori and its application in biomaterials and biomedicines. Mater Sci Eng C Mater Biol Appl. 2016;61:940‐952. 10.1016/j.msec.2015.12.082 26838924

[52] Altman GH , Diaz F , Jakuba C , et al. Silk‐based biomaterials. Biomaterials. 2003;24(3):401‐416. 12423595 10.1016/s0142-9612(02)00353-8

[53] Soong HK , Kenyon KR . Adverse reactions to virgin silk sutures in cataract surgery. Ophthalmology. 1984;91(5):479‐483. 10.1016/S0161-6420(84)34273-7 6377167

[54] Vollrath F . Strength and structure of spiders' silks. J Biotechnol. 2000;74(2):67‐83. 11763504 10.1016/s1389-0352(00)00006-4

[55] van Beek JD , Hess S , Vollrath F , Meier BH . The molecular structure of spider dragline silk: folding and orientation of the protein backbone. Proc Natl Acad Sci U S A. 2002;99(16):10266‐10271. 10.1073/pnas.152162299 12149440 PMC124902

[56] Roloff F , Strauss S , Vogt PM , Bicker G , Radtke C . Spider silk as guiding biomaterial for human model neurons. Biomed Res Int. 2014;2014:906819. 10.1155/2014/906819 24949480 PMC4052499

[57] Kuhbier JW , Allmeling C , Reimers K , et al. Interactions between spider silk and cells–NIH/3T3 fibroblasts seeded on miniature weaving frames. PLoS One. 2010;5(8):e12032. 10.1371/journal.pone.0012032 20711495 PMC2918503

[58] Kuhbier JW , Reimers K , Kasper C , et al. First investigation of spider silk as a braided microsurgical suture. J Biomed Mater Res B Appl Biomater. 2011;97B(2):381‐387. 10.1002/jbm.b.31825 21432995

[59] Radtke C , Allmeling C , Waldmann KH , et al. Spider silk constructs enhance axonal regeneration and remyelination in long nerve defects in sheep. PLoS One. 2011;6(2):e16990. 10.1371/journal.pone.0016990 21364921 PMC3045382

[60] Salehi S , Koeck K , Scheibel T . Spider silk for tissue engineering applications. Molecules. 2020;25(3). 10.3390/molecules25030737 PMC7037138 32046280

[61] Heidebrecht A , Scheibel T . Chapter Four—Recombinant production of spider silk proteins. 2013:115‐153. 10.1016/B978-0-12-407679-2.00004-1 23415154

[62] Ling S , Qin Z , Li C , Huang W , Kaplan DL , Buehler MJ . Polymorphic regenerated silk fibers assembled through bioinspired spinning. Nat Commun. 2017;8(1):1387. 10.1038/s41467-017-00613-5 29123097 PMC5680232

[63] Zhou Y , Shen Q , Lin Y , Xu S , Meng Q . Evaluation of the potential of chimeric spidroins/poly(L‐lactic‐co‐ε‐caprolactone) (PLCL) nanofibrous scaffolds for tissue engineering. Mater Sci Eng C Mater Biol Appl. 2020;111:110752. 10.1016/j.msec.2020.110752 32279827

[64] Zhu B , Li W , Lewis RV , Segre CU , Wang R . E‐spun composite fibers of collagen and dragline silk protein: fiber mechanics, biocompatibility, and application in stem cell differentiation. Biomacromol. 2015;16(1):202‐213. 10.1021/bm501403f PMC4294589 25405355

[65] Jacobsen MM , Li D , Gyune Rim N , Backman D , Smith ML , Wong JY . Silk‐fibronectin protein alloy fibers support cell adhesion and viability as a high strength, matrix fiber analogue. Sci Rep. 2017;7(1):45653. 10.1038/srep45653 28378749 PMC5381220

[66] Dinis TM , Vidal G , Jose RR , et al. Complementary effects of two growth factors in multifunctionalized silk nanofibers for nerve reconstruction. PLoS One. 2014;9(10):e109770. 10.1371/journal.pone.0109770 25313579 PMC4196919

[67] Steins A , Dik P , Müller WH , et al. In vitro evaluation of spider silk meshes as a potential biomaterial for bladder reconstruction. PLoS One. 2015;10(12):e0145240. 10.1371/journal.pone.0145240 26689371 PMC4687005

[68] Pawar K , Welzel G , Haynl C , Schuster S , Scheibel T . Recombinant spider silk and collagen‐based nerve guidance conduits support neuronal cell differentiation and functionality in vitro. ACS Applied Bio Materials. 2019;2(11):4872‐4880. 10.1021/acsabm.9b00628 35021487

[69] Mandal BB , Kundu SC . Cell proliferation and migration in silk fibroin 3D scaffolds. Biomaterials. 2009;30(15):2956‐2965. 10.1016/j.biomaterials.2009.02.006 19249094

[70] Baron‐Van Evercooren A , Gansmüller A , Gumpel M , Baumann N , Kleinman HK . Schwann cell differentiation in vitro: extracellular matrix deposition and interaction. Dev Neurosci. 1986;8(3):182‐196. 10.1159/000112252 3021428

[71] Parrinello S , Napoli I , Ribeiro S , et al. EphB signaling directs peripheral nerve regeneration through Sox2‐dependent Schwann cell sorting. Cell. 2010;143(1):145‐155. 10.1016/j.cell.2010.08.039 20869108 PMC3826531

[72] Obremski VJ , Wood PM , Bunge MB . Fibroblasts promote Schwann cell basal lamina deposition and elongation in the absence of neurons in culture. Dev Biol. 1993;160(1):119‐134. 10.1006/dbio.1993.1291 8224529

[73] Wang ML , Rivlin M , Graham JG , Beredjiklian PK . Peripheral nerve injury, scarring, and recovery. Connect Tissue Res. 2019;60(1):3‐9. 10.1080/03008207.2018.1489381 30187777

[74] European Parliament CotEU . Directive 2010/63/EU of the European Parliament and of the Council of 22 September 2010 on the protection of animals used for scientific purposes (text with EEA relevance). Official Journal of the European Union. 2010;53(L 276):33‐79.

[75] Haertinger M , Weiss T , Mann A , Tabi A , Brandel V , Radtke C . Adipose stem cell‐derived extracellular vesicles induce proliferation of schwann cells via internalization. Cells. 2020;9(1):163. 10.3390/cells9010163 31936601 PMC7016740

[76] Weiss T , Taschner‐Mandl S , Ambros PF , Ambros IM . Detailed protocols for the isolation, culture, enrichment and immunostaining of primary human Schwann cells. Methods Mol Biol. 2018;1739:67‐86. 29546701 10.1007/978-1-4939-7649-2_5

[77] Lee S‐I , Levine J . Isolation and growth of adult mouse dorsal root ganglia neurons. Bio‐protocol. 2015;5(18):e1601. 10.21769/BioProtoc.1601

[78] Malin SA , Davis BM , Molliver DC . Production of dissociated sensory neuron cultures and considerations for their use in studying neuronal function and plasticity. Nat Protoc. 2007;2(1):152‐160. 10.1038/nprot.2006.461 17401349

[79] Bretz F , Hothorn T , Westfall P . Multiple Comparison Using R. New York, NY: CRC Press, Taylor & Francis Group; 2016:205 p.

[80] Chen MB , Zhang F , Lineaweaver WC . Luminal fillers in nerve conduits for peripheral nerve repair. Ann Plast Surg. 2006;57(4):462‐471. 10.1097/01.sap.0000237577.07219.b6 16998343

[81] Magaz A , Faroni A , Gough JE , Reid AJ , Li X , Blaker JJ . Bioactive silk‐based nerve guidance conduits for augmenting peripheral nerve repair. Adv Healthc Mater. 2018;7(23):e1800308. 10.1002/adhm.201800308 30260575

[82] Eldridge CF , Bunge MB , Bunge RP . Differentiation of axon‐related Schwann cells in vitro: II. Control of myelin formation by basal lamina. J Neurosci. 1989;9(2):625‐638. 10.1523/jneurosci.09-02-00625.1989 2918381 PMC6569783

[83] Martini R , Schachner M . Immunoelectron microscopic localization of neural cell adhesion molecules (L1, N‐CAM, and myelin‐associated glycoprotein) in regenerating adult mouse sciatic nerve. J Cell Biol. 1988;106(5):1735‐1746. 10.1083/jcb.106.5.1735 2453520 PMC2115039

[84] Abercrombie M . Contact inhibition in tissue culture. Vitro. 1970;6(2):128‐142. 10.1007/bf02616114 4943054

[85] Yang DP , Zhang DP , Mak KS , Bonder DE , Pomeroy SL , Kim HA . Schwann cell proliferation during Wallerian degeneration is not necessary for regeneration and remyelination of the peripheral nerves: axon‐dependent removal of newly generated Schwann cells by apoptosis. Mol Cell Neurosci. 2008;38(1):80‐88. 10.1016/j.mcn.2008.01.017 18374600 PMC2440648

[86] Hoke A . Mechanisms of disease: what factors limit the success of peripheral nerve regeneration in humans? Nat Clin Pract Neurol. 2006;2(8):448‐454. 10.1038/ncpneuro0262 16932603

[87] Fu SY , Gordon T . The cellular and molecular basis of peripheral nerve regeneration. Mol Neurobiol. 1997;14(1–2):67‐116. 10.1007/bf02740621 9170101

[88] Sulaiman W , Gordon T . Neurobiology of peripheral nerve injury, regeneration, and functional recovery: from bench top research to bedside application. Ochsner J. 2013;13(1):100‐108. 23531634 PMC3603172

[89] Naghilou A , Pöttschacher L , Millesi F , et al. Correlating the secondary protein structure of natural spider silk with its guiding properties for Schwann cells. Mater Sci Eng, C. 2020;116:111219. 10.1016/j.msec.2020.111219 32806225

[90] Webb K , Hlady V , Tresco PA . Relative importance of surface wettability and charged functional groups on NIH 3T3 fibroblast attachment, spreading, and cytoskeletal organization. J Biomed Mater Res. 1998;41(3):422‐430. 10.1002/(sici)1097-4636(19980905)41:3<422::aid-jbm12>3.0.co;2-k 9659612 PMC2632339

[91] De Rosa M , Carteni' M , Petillo O , et al. Cationic polyelectrolyte hydrogel fosters fibroblast spreading, proliferation, and extracellular matrix production: Implications for tissue engineering. J Cell Physiol. 2004;198(1):133‐143. 10.1002/jcp.10397 14584053

[92] Leal‐Egaña A , Scheibel T . Interactions of cells with silk surfaces. J Mater Chem. 2012;22(29):14330‐14336. 10.1039/C2JM31174G

[93] Petzold J , Aigner TB , Touska F , Zimmermann K , Scheibel T , Engel FB . Surface features of recombinant spider silk protein eADF4(κ16)‐made materials are well‐suited for cardiac tissue engineering. Adv Func Mater. 2017;27(36):1701427. 10.1002/adfm.201701427

[94] Wohlrab S , Müller S , Schmidt A , et al. Cell adhesion and proliferation on RGD‐modified recombinant spider silk proteins. Biomaterials. 2012;33(28):6650‐6659. 10.1016/j.biomaterials.2012.05.069 22727466

[95] Kluge JA , Rabotyagova O , Leisk GG , Kaplan DL . Spider silks and their applications. Trends Biotechnol. 2008;26(5):244‐251. 10.1016/j.tibtech.2008.02.006 18367277

[96] Bini E , Foo CW , Huang J , Karageorgiou V , Kitchel B , Kaplan DL . RGD‐functionalized bioengineered spider dragline silk biomaterial. Biomacromol. 2006;7(11):3139‐3145. 10.1021/bm0607877 17096543

[97] Widhe M , Johansson U , Hillerdahl CO , Hedhammar M . Recombinant spider silk with cell binding motifs for specific adherence of cells. Biomaterials. 2013;34(33):8223‐8234. 10.1016/j.biomaterials.2013.07.058 23916396

[98] Dun XP , Parkinson DB . Visualizing peripheral nerve regeneration by whole mount staining. PLoS One. 2015;10(3):e0119168. 10.1371/journal.pone.0119168 25738874 PMC4349735

[99] Lin Y‐C , Ramadan M , Hronik‐Tupaj M , et al. Spatially controlled delivery of neurotrophic factors in silk fibroin‐based nerve conduits for peripheral nerve repair. Ann Plast Surg. 2011;67(2):147‐155. 10.1097/SAP.0b013e3182240346 21712696

[100] Madhusudanan P , Raju G , Shankarappa S . Hydrogel systems and their role in neural tissue engineering. J R Soc Interface. 2020;17(162):20190505. 10.1098/rsif.2019.0505 31910776 PMC7014813

[101] Lee H , McKeon RJ , Bellamkonda RV . Sustained delivery of thermostabilized chABC enhances axonal sprouting and functional recovery after spinal cord injury. Proc Natl Acad Sci USA. 2010;107(8):3340‐3345. 10.1073/pnas.0905437106 19884507 PMC2840440

[102] Yousefi F , Lavi Arab F , Nikkhah K , Amiri H , Mahmoudi M . Novel approaches using mesenchymal stem cells for curing peripheral nerve injuries. Life Sci. 2019;221:99‐108. 10.1016/j.lfs.2019.01.052 30735735

[103] Di Summa PG , Schiraldi L , Cherubino M , et al. Adipose derived stem cells reduce fibrosis and promote nerve regeneration in rats. Anat Rec (Hoboken). 2018;301(10):1714‐1721. 10.1002/ar.23841 29710394 PMC6667902

[104] Hood B , Levene HB , Levi AD . Transplantation of autologous Schwann cells for the repair of segmental peripheral nerve defects. Neurosurg Focus. 2009;26(2):E4. 10.3171/foc.2009.26.2.E4 19435444

[105] Sinis N , Schaller H‐E , Schulte‐Eversum C , et al. Nerve regeneration across a 2‐cm gap in the rat median nerve using a resorbable nerve conduit filled with Schwann cells. J Neurosurg. 2005;103(6):1067‐1076. 10.3171/jns.2005.103.6.1067 16381194

[106] Choe G , Park J , Park H , Lee JY . Hydrogel biomaterials for stem cell microencapsulation. Polymers (Basel). 2018;10(9). 10.3390/polym10090997 PMC6403586 30960922

