# Supplementary material for: Defining the regenerative effects of native spider silk fibers on primary Schwann cells, sensory neurons, and nerve‐associated fibroblasts
Source: FASEB J. 2020 Nov 19;35(2):e21196. doi: 10.1096/fj.202001447R (PMC7894153; doi:10.1096/fj.202001447R)
Supplement: Supplementary file 1 — Table S1 [file FSB2-35-e21196-s002.docx]

**Supplementary Table 1: Primary and secondary antibodies**

| **Primary Antibodies** | | | | |
| --- | --- | --- | --- | --- |
| **Antigen** | **species** | **Dilution** | **Company** | **Comment** |
| S100 | rabbit | 1:200 | DAKO, #Z0311 | Permeabilization,  o.n. incubation at 4°C |
| Vimentin  (VIME) | chicken | 1:300 | ThermoFisher, #PA1-10003 | Permeabilization  o.n. incubation at 4°C |
| β-3-tubulin  (TUJ1) | mouse | 1:300 | Santa Cruz, sc-51670 | Permeabilization  o.n. incubation at 4°C |
| THY1 | mouse | 1:50 | Santa Cruz, sc-53116 | No permeabilization  o.n. incubation at 4°C |
| P75^NTR^ (D4B3) XP  (NGFR) | rabbit | 1:400 | CellSignaling. #8238S | No permeabilization  o.n. incubation at 4°C |
| **Secondary Antibodies** | | | | |
| **Fluorophore** | **Target** | **Dilution** | **Company** | **Comment** |
| AF488P | rabbit | 1:800 | Invitrogen, #A11029 | 1 h incubation, RT |
| AF594 | mouse | 1:400 | ThermoFisher, #T2767 | 1 h incubation, RT |
| DL650 | chicken | 1:400 | ThermoFisher, #A11056 | 1 h incubation, RT |
| AF488 | mouse | 1:400 | ThermoFisher, #A-11029 | 1 h incubation, RT |
